# The in-stream renewable energy potential of rivers for remote communities in the global Arctic

**DOI:** 10.1038/s41467-026-74292-6

**Published:** 2026-06-16

**Authors:** Katelyn Kirby, Colin D. Rennie, Ioan Nistor, Cameron Johnstone, Stephanie Ordoñez-Sánchez, Song Fu, Julien Cousineau, Heather Shilton

**Affiliations:** 1 Department of Civil Engineering, University of Ottawa, Ottawa, ON Canada; 2 Ocean, Coastal and River Engineering Research Centre, National Research Council Canada, Ottawa, ON Canada; 3 Department of Mechanical and Aerospace Engineering, University of Strathclyde, Glasgow, UK; 4 Nunavut Nukkiksautiit Corporation, Iqaluit, NU Canada

**Keywords:** Energy supply and demand, Energy access, Climate-change mitigation

## Abstract

Many Arctic communities rely on diesel-generated electricity, resulting in high costs, environmental impacts, and limited development opportunities. Hydrokinetic energy (HKE), which generates power from flowing water without dams, offers a lower-impact renewable energy alternative to conventional hydropower. Here we identify feasible Arctic communities with suitable river conditions for HKE development and highlight regions with the greatest potential. Using a detailed case study of the Iqaluit Kuunga River in Nunavut, Canada, alongside a global assessment, we identify 325 Arctic communities suitable for HKE deployment. These sites represent a theoretical energy potential of 4,626 megawatt-hours per square metre of turbine area annually under open water conditions. Feasibility varies with the local resource availability, proximity to river sites, and projected diesel offset. Together, these results highlight the potential for HKE to support cleaner, community-based energy systems, and the datasets provide a foundation for targeted renewable energy transitions in Arctic communities.

## Abstract (web summary)

Diesel use in remote Arctic communities could be reduced with hydrokinetic river turbines. The study identifies 325 viable communities globally and confirms feasibility in Nunavut, Canada, highlighting strong potential for cleaner local energy.

## Introduction

The growing push to develop renewable energy (RE) is fuelled by extensive evidence of the negative environmental impacts of fossil fuel-based energy sources^1^. As of 2021, 79% of Arctic communities in North America (Canada, Greenland, and Alaska, United States of America) still relied on diesel for electricity^2^. This reliance has caused environmental degradation and hindered community development. In Canadian Indigenous communities, diesel-based electricity generation has compromised service quality and impeded innovation^3^.

All Arctic regions, reliant on seasonal navigation and winter roads, face logistical challenges and negative consequences of delivering diesel^4^. Black carbon from fossil fuels accelerates local ice and snow melt, reducing the Arctic’s reflectivity^4,5^ and altering historical ice stability patterns. These changes lead to increased risk for hunters traveling to traditional hunting areas. The risk of changing sea ice patterns and its impact on subsistence activities in northern communities has not yet been quantified. Studies that have quantified the impact of black carbon on snow and ice albedo have approximated a 20% albedo reduction during glacier melt season in the Tibetan Plateau^6^. Despite the lack of quantified risk in literature, these changes are common knowledge in northern communities^7,8^, with climate adaptation tools—such as SmartICE^9^—being developed to aid hunters to travel on sea ice safely. Further, market fluctuations tied to fuel shipping and storage have only added to the complexity of development^4^. Thus, communities are seeking alternatives due to the high costs, environmental risks, and long-term sustainability concerns of diesel^10^.

The Arctic is home to a growing population of approximately four million people^11^. Yet, its vast geography presents energy challenges due to mountains, tundra, and extreme cold weather conditions^2^. Key challenges for energy availability in the Arctic include costly transportation, limited infrastructure, and environmental and health risks^12^. In Canada, 65% of energy is produced from renewable sources, but this rarely benefits remote Arctic communities disconnected from the southern grid^13^. Energy costs in Arctic Canada are also among the nation’s highest^14^. In Nunavut, 20% of the government’s annual budget subsidises diesel-based electricity^15^, diverting funds from pressing needs, such as mental health, housing, and food security. Similarly, in Arctic Russia, monopolies and high electricity prices strain households financially, where prices reach 600 rubles (~7.56 United States Dollars or USD) per kWh compared to the national average of 3 rubles per kWh (~0.04 USD)^4^. Existing energy systems in these regions operate at a deficit, forcing utilities to pass costs onto consumers^10^. With energy availability being critical for survival, especially in low-income communities—with high unemployment rates, poorer housing conditions, fewer opportunities, and reduced access to services—in extreme environments, addressing these barriers is essential for improving Arctic energy sustainability^2^.

Community studies in Nunavut, Canada, show strong support for RE adoption, with little backing for continued diesel use^16–18^. Hydropower already plays a large role, accounting for 44% of global Arctic electricity compared to 16% globally^2^. However, hydropower development in Arctic Canada has faced opposition due to environmental and social concerns, including the flooding of Indigenous lands and treaty violations^13,15,19^.

Hydrokinetic energy (HKE) presents a promising alternative to traditional hydropower by harnessing run-of-river currents without requiring dams. Unlike conventional hydropower, hydrokinetic turbines minimize impact to river ecology^20^ and sediment transport^21–23^. The Arctic’s HKE is notable, with rivers (excluding Greenland) expected to produce 2000 terawatt-hours (TWh) annually^24,25^. HKE viability has been demonstrated in Igiugig, Alaska, where a single turbine reduced diesel use by 50% during peak demand and reduced emissions by 98.7%^26^. Additionally, small hydrokinetic systems are relatively simple to install and maintain, making them a practical solution for remote Arctic communities^27^. However, the feasibility in Arctic rivers with long ice cover requires examination. To continue to drive this technology forward, further studies on HKE are necessary, focusing on optimal sizing, reliability, and policy support^27^.

To support the development of policies for the exploration and development of HKE in the Arctic, a feasibility assessment of HKE in the Arctic is presented, specifically examining the Iqaluit Kuunga River in Nunavut, Canada. Additionally, HKE resources are analysed broadly across the global Arctic to identify strategic locations for deployment, considering technical, community, and socio-economic factors. By advancing HKE research, this study aims to support sustainable and locally beneficial energy solutions for Arctic communities.

## Results

### Hydrokinetic energy feasibility assessment of an Arctic river

**Fig. 1:**
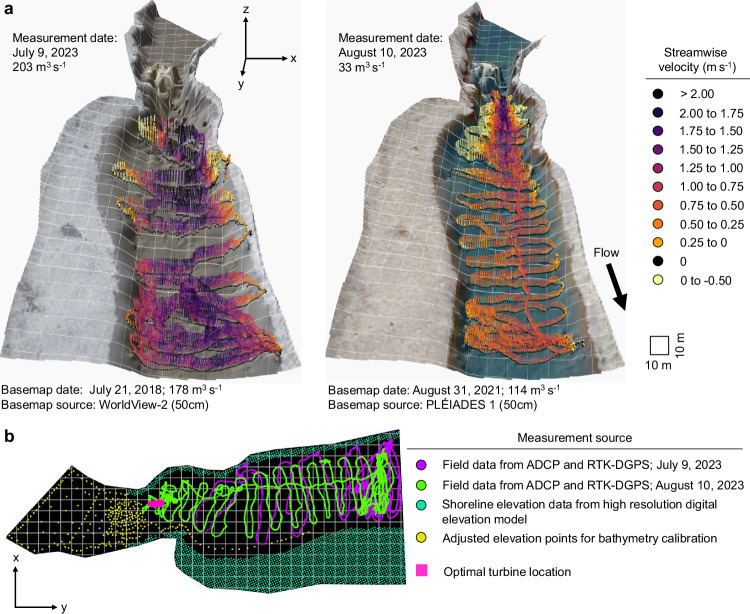
Field data collected at the Iqaluit Kuunga River, Nunavut, Canada, to support HKE resource estimation and turbine staging. **a** Acoustic Doppler current profiler measurements under two distinct flow conditions. Satellite imagery base maps were purchased from WorldView-2 (DigitalGlobe) and PLÉIADES 1 (Planet). Includes copyrighted material of DigitalGlobe, Inc. © CNES (2021), Licensed by Planet Labs Geomatics Corp. **b** Spatial coverage and sources of riverbed bathymetry (from ADCP and high-resolution digital elevation model), water surface elevation (from ADCP), and three-dimensional flow velocity data (from ADCP). Upstream riverbed elevation points were adjusted near the width constriction and elevation drop to calibrate the hydrodynamic model, aligning simulated velocities with field measurements. The optimal turbine location (OTL) is shown in pink, as determined by water depth and flow velocity thresholds. Figure created using ArcGIS Pro 3.3^94^.

**Fig. 2:**
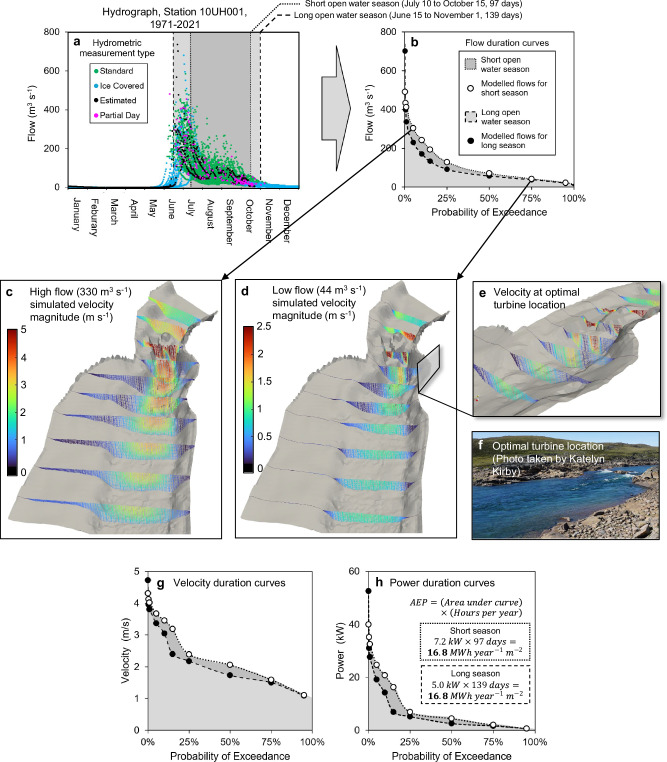
Hydrodynamic modeling and HKE analysis for the Iqaluit Kuunga River site. **a** Site hydrograph from 51 years of hydrometric data located 5 km downstream from the site, also indicating the two scenarios used to calculate the HKE: a typical short open water season and a typical long open water season based on observations of break-up and freeze-up times from satellite imagery and the hydrometric data. **b** The two flow duration curves generated from only the standard hydrometric measurement data (ice-covered, estimated, and partial-day measurements removed) represent a short open water season and a long open water season. **c** Simulated velocity flow field for a high flow condition (330 m^3^ s^−1^) from the calibrated (root mean squared error, RMSE, of 0.34 m s^−1^ and mean absolute percent error, MAPE, of 22.0%) and validated (RMSE of 0.26 m s^−1^ and MAPE of 10.5%) hydrodynamic model. **d** Simulated velocity flow field for the low flow condition (44 m^3^ s^−1^). **e** Simulated velocity flow field at the optimal turbine location for the low flow condition (44 m^3^ s^−1^), similar to (**f**) the low flow condition measured on August 10, 2023 (33 m^3^ s^−1^; Fig. 1b), shown for visual comparison. **g** The two velocity duration curves for the site, based on the simulated velocity data. **h** The two power duration curves for the site and associated annual energy production (AEP) for each of the two open water conditions.

To address the knowledge gap on HKE viability in the Arctic, a case study was conducted on the Iqaluit Kuunga River (Sylvia Grinnell River), located 5 km northwest of Iqaluit, Nunavut, Canada (63.787°N, −68.622°W). With an annual average air temperature of −8.6 °C^28^, the site is colder than 97.5% of the 3129 northern communities identified in this study above 55°N. The HKE resource was assessed using field data (Fig. 1) and hydrodynamic modeling (Fig. 2), confirming the site’s technical feasibility for turbine deployment. Theoretical energy potential was estimated at 16.8 MW h per year per m^2^ of turbine capture area, with velocity consistently above 1.0 m s^−1^, even in late summer low flow conditions, a value often cited as an indicator of HKE feasibility^29^.

Given Nunavut’s per capita electricity use of 6.03 megawatt-hours (MWh) per year^30^, a 10 m^2^ Savonius turbine with an optimized coefficient of power of 0.45^29,31^ could potentially supply electricity to 12–13 people. Because turbines are generally deployed in arrays^32^, this estimate is conservative. Using the ORPC RivGen^26,33^ turbine power curve, the site’s AEP is projected at 95–105 MW h per year during open water months for a single turbine, considering a 1 m s^−1^ cut-in and 3.5 m s^−1^ cut-out velocity. Adjusting for operational days, this energy yield is comparable to the Kvichak River installation in Igiugig, Alaska, which produces 349 MW h per year and offsets 60–90% of community diesel use^26,33^.

To derive these estimates of HKE resource at the study site, field data were collected with an acoustic Doppler current profiler (ADCP) mounted on a remote-controlled boat (Fig. 1). This provided three-dimensional velocity, bathymetry, and water surface elevation. Further information on the field data collection and processing is available in the “Methods”. These data identified the optimal turbine location (OTL) (Fig. 1b) to be directly downstream of a river width constriction. Here, flow velocity is increased by lateral narrowing and an elevation drop, creating a scour hole of 6 m (high flow) to 3 m (low flow). The combination of increased streamwise velocity and greater depth makes this site ideal for turbine deployment. Similar features may indicate suitability for HKE development elsewhere.

At the Iqaluit Kuunga site, the river freezes completely to the bed, as shown in the site hydrograph (Fig. 2a); thus, HKE was calculated only for the open water season. Ice conditions would necessitate seasonal turbine removal and reinstallation to mitigate damage, resulting in less energy production and higher labor costs for this site. In contrast, the community of Igiugig, Alaska, with a higher mean annual temperature of 1.9 °C, has reported no turbine ice issues during operation over two winters^34^. Logistical and engineering challenges of this nature were not further explored in this study, as these challenges will vary at each river location.

The hydrodynamic model, calibrated and validated with ADCP data (Fig. 1), simulated a range of representative flows at the site. Two flow duration curves (FDCs) (Fig. 2b), derived from the site hydrograph, informed the selection of twenty flow conditions, ten each for long and short open water seasons. Velocity fields (Fig. 2c) were simulated for these conditions to estimate OTL velocities (Figs. 1b and 2c) and generate corresponding velocity duration curves (VDCs) (Fig. 2d). Simulations showed that velocities at the OTL consistently exceeded 1.0 m s^−1^ during open water periods. The annual energy production (AEP), calculated from the power duration curves (PDCs) (Fig. 2e), was similar for both season lengths due to higher early-season flows in the short season, likely driven by concentrated melt events. However, ice presence during these high flows may hinder turbine deployment, reducing actual energy capture relative to theoretical estimates.

### Technical in-stream potential near remote communities in Canada

Building on the Iqaluit Kuunga River case study, the analysis was extended to evaluate HKE potential across the Canadian Arctic and sub-Arctic. Each remote community was assessed using the Canadian River Hydrokinetic Energy (CRHE)^35^ and Remote Communities Energy (RCE)^36^ databases, which provide detailed, nationally sourced data on hydrokinetic potential and electricity generation profiles, respectively, including current electricity source and amount for the 178 remote communities in the RCE database. More details on this process are provided in the “Methods” section, and the community HKE estimates and diesel offset values are provided in Supplementary Data 1.

**Fig. 3:**
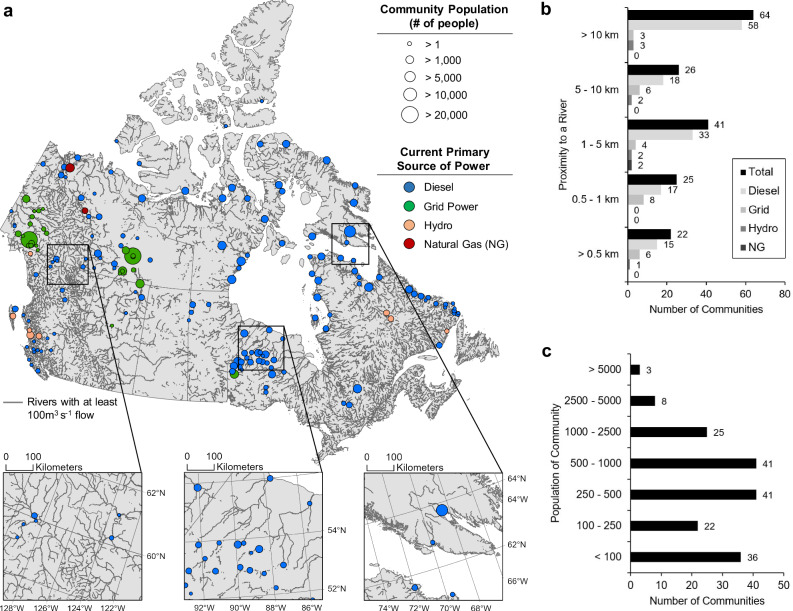
Remote Arctic and sub-Arctic communities in Canada with river proximity and population distributions. **a** Remote communities in Canada, most of which are First Nations or Inuit (28.7% Inuit, 61.8% First Nations, 4.5% indigenous collective, 5.1% non-indigenous), and their primary source of electricity generation, as well as the community population. **b** Community proximity to rivers and current primary power generation. Communities within 10 km of a river and currently on diesel-based electricity generation are most likely to be feasible for and benefit from HKE. **c** The division of communities into population categories. All geospatial layers (Canada outline, river centerlines, and remote community locations) shown in this figure are distributed under the Government of Canada Open Government Licence. Figure created using ArcGIS Pro 3.3^94^.

Figure 3a–c show community locations, proximity to rivers, and population distribution, respectively. Most communities (64%) are within 10 km of a river, and 79% of communities rely primarily on diesel for electricity. Few communities have populations exceeding 1000 people, and the median inter-community distance is 77 km (IQR 47–122 km). An estimated 17,500 km of transmission lines would be needed to interconnect all communities, indicating the practicality of community-based electricity generation on local microgrids as opposed to centralized grid integration^37^.

**Fig. 4:**
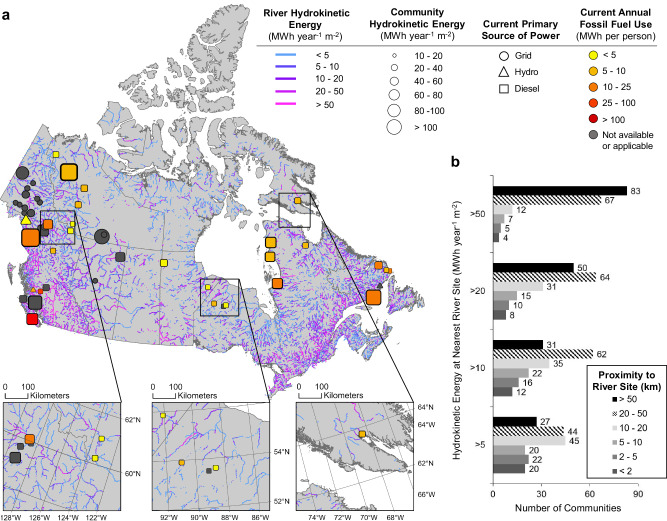
Remote Canadian communities identified as feasible for HKE development. **a** The Canadian River Hydrokinetic Energy (CRHE) database, and remote communities in Canada within 10 km of a 10 MW h year^−1^ m^−2^ hydrokinetic river site. Communities are symbolized by the estimated amount of river HKE available near the community (size), the current main electricity source (shape), and the community’s current annual per capita fossil fuel use (color). The communities currently reliant on diesel with a high nearby river HKE resource are the most promising for turbine deployment. **b** A bar graph detailing the distribution of communities between their distance to the nearest feasible hydrokinetic river site and the amount of potential energy at the nearest river site. All geospatial layers (Canada outline, river centerlines, and remote community locations) shown in this figure are distributed under the Government of Canada Open Government Licence. Figure created using ArcGIS Pro 3.3^94^.

Figure 4a highlights communities within 10 km of river sites with HKE exceeding 10 MW h year^−1^ m^−2^. Because the CRHE dataset represents rivers with flows greater than 100 m^3^ s^−1^, there is still a small likelihood that communities not included in Fig. 4a may still be feasible for HKE.

A total theoretical maximum AEP of 1770 MW h year^−1^ m^−2^ is estimated from the 50 remote communities identified to be feasible. Using fossil fuel generation data from the RCE database^36^, ten communities could achieve greater than 20% diesel offset with a single turbine, and fifteen could achieve greater than 5%. Among the 50 identified communities, the median diesel offset from one turbine was 6.7% (IQR 3.1–27.8%).

No correlation was found between the mean annual air temperature over the 1994–2024 period and the per-capita fossil fuel-based electricity usage for the 178 communities in the RCE database (*R*^2^ = 0.076), excluding communities with major industrial activity (i.e., mining, forestry, etc.). Thus, despite a 23 °C range in mean community temperatures (median −3.5 °C; IQR −7.2 to −0.35 °C), energy use was not temperature-dependent. Similarly, no correlation was found between temperature and diesel offset from HKE (*R*² = 0.040), indicating that, given suitable river conditions (i.e., power, ice, and installation conditions), colder communities can benefit from HKE just as much as warmer ones.

### Global Arctic technical in-stream potential near communities

**Fig. 5:**
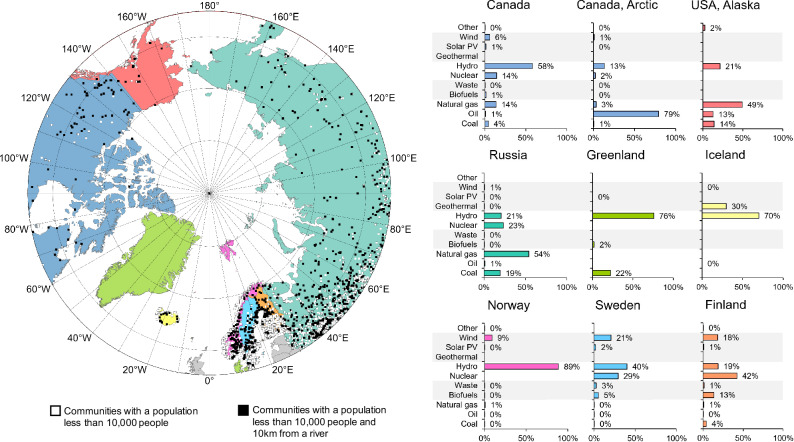
Communities with a population of less than 10,000 people north of 55°N latitude. Communities located within 10 km of a river are indicated in black. Each country considered in this analysis is listed to the right with the percent of total electricity in the countries also illustrated. The comparison between the Canadian Arctic and wider Canada illustrates the disparity between northern communities’ electricity sources and the rest of the country. Community locations were obtained from the Geographic Names Server developed by the National Geospatial-Intelligence Agency (unrestricted), and global boundary shapefiles were sourced from Natural Earth public domain datasets. Figure created using ArcGIS Pro 3.3^94^.

**Fig. 6:**
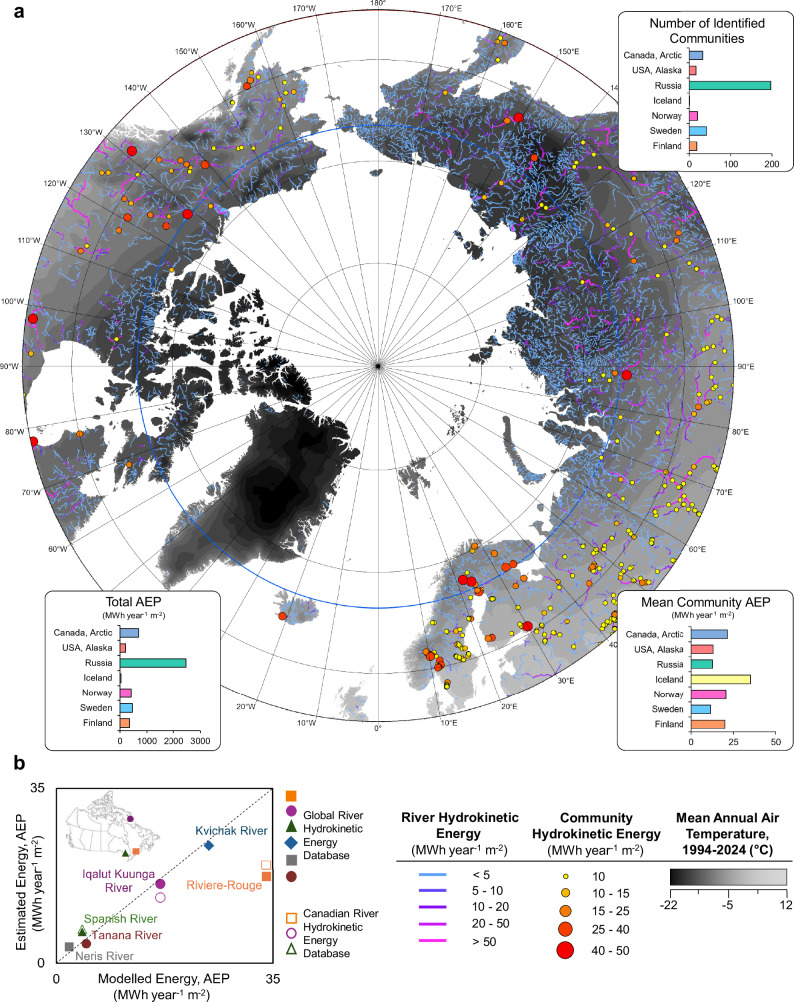
Communities identified as feasible for HKE development in the global Arctic. **a** Estimates of river HKE derived from global datasets, locations of communities within 10 km of a river site with at least 10 MW h year^−1^ m^−2^ estimated, and mean annual air temperature from daily temperature estimates in the 1994–2024 period. Also illustrated is the total AEP for all identified communities in each of the eight countries included in this analysis (Greenland excluded as no feasible communities were found), the total number of communities identified in each country, and the mean community AEP for each country. **b** Validation of the HKE estimates from the CRHE database and the global database at three locations where the AEP was modeled using field measurements and hydrodynamic models, and another three locations from independent hydrokinetic resource assessment studies^26,92,93^. Due to the limited data collection for the study on the Tanana River, Alaska^92^, AEP for this location is presented as a monthly value (MW h month^−1^ m^−2^ rather than MW h year^−1^ m^−2^) to provide comparable values. Community locations were obtained from the Geographic Names Server developed by the National Geospatial-Intelligence Agency (unrestricted), global boundary shapefiles were sourced from Natural Earth public domain datasets, and the Canada shapefile is distributed under the Government of Canada Open Government Licence. World river centerlines were derived from the GRADES dataset^84^. Figure created using ArcGIS Pro 3.3^94^.

The HKE feasibility analysis was expanded to global Arctic and sub-Arctic communities, where available data is less detailed than in Canada. Figure 5 illustrates Arctic and sub-Arctic communities with populations under 10,000 to focus this analysis on remote, smaller communities. River energy estimates, shown in Fig. 6 and provided in Supplementary Data 3, were derived from global databases (see “Methods”) to identify communities suitable for hydrokinetic development. Of the 3129 communities identified, 1007 are within 10 km of a river. Figure 5 includes national electricity source distributions from the International Energy Agency^38^. The contrast between Arctic and national energy profiles, illustrated for Canada, demonstrates that national statistics often fail to capture conditions in remote regions. Nonetheless, these distributions provide useful context by highlighting countries with high fossil fuel use that may be well-suited for HKE transitions.

The total AEP for global Arctic communities was estimated at 4626 MW h year^−1^ m^−2^ across 325 identified communities (Fig. 6a). The community HKE estimates are provided in Supplementary Data 2. For Canada, the global approach yielded 684 MW h year^−1^ m^−2^, compared to 1326 MW h year^−1^ m^−2^ from the national assessment. Similarly, 31 feasible Canadian communities were identified globally versus 50 nationally. This discrepancy reflects differences in data resolution, with the CHRE database incorporating more detailed river channel dimensions and a more comprehensive river network. Consequently, the global assessment likely underestimates AEP, though both datasets showed good agreement with validation data at three sites (Fig. 6b).

Russia had the highest total AEP at 2470 MW h year^−1^ m^−2^ across 197 communities, while Iceland had the lowest, with one community at 35 MW h year^−1^ m^−2^. Country-level AEP totals and the number of identified communities are illustrated in Fig. 6a. Despite its high total, Russia’s average AEP per community (12.5 MW h year^−1^ m^−2^) was the second lowest, ahead of Sweden (11.3 MW h year^−1^ m^−2^). Iceland had the highest AEP per community (35.0 MW h year^−1^ m^−2^) for the one community identified as feasible. The next highest were Canada (21.4 MW h year^−1^ m^−2^), Norway (20.5 MW h year^−1^ m^−2^), and Finland (19.8 MW h year^−1^ m^−2^). Similar to the Canadian assessment, most feasible communities were located near river sites with sudden changes in river dimensions from local topography (e.g., bedrock width constrictions or steep mountain gradients)^35^.

The Arctic is warming faster than the global average, driving rapid changes in its hydrological cycle^39^ with implications for HKE availability and distribution^40^. A transition from a snow- to rain-dominated regime, along with increased total precipitation, is expected^39^. This shift will likely alter the shape and distribution of FDCs (Fig. 2b)^40^, reducing melt-driven flows and increasing summer and fall precipitation^39^. Historical data from the Eurasian Arctic indicate increased subsurface connectivity due to permafrost thaw, affecting rainfall-runoff dynamics and the mechanisms behind how rainfall is routed to rivers^40^. Concurrently, channel morphology may change as permafrost and ice, key factors in channel stability, diminish^41^. River ice dynamics increase sediment movement both directly and by increasing vertical channel growth^41,42^; thus, it is expected that channels will become more mobile. These changes to precipitation, rainfall-runoff dynamics, and channel stability may both positively and negatively impact the magnitude and spatiotemporal distribution of HKE across the global Arctic in the future.

### Socioeconomic realities for hydrokinetic and renewable energy development in the Arctic

**Fig. 7:**
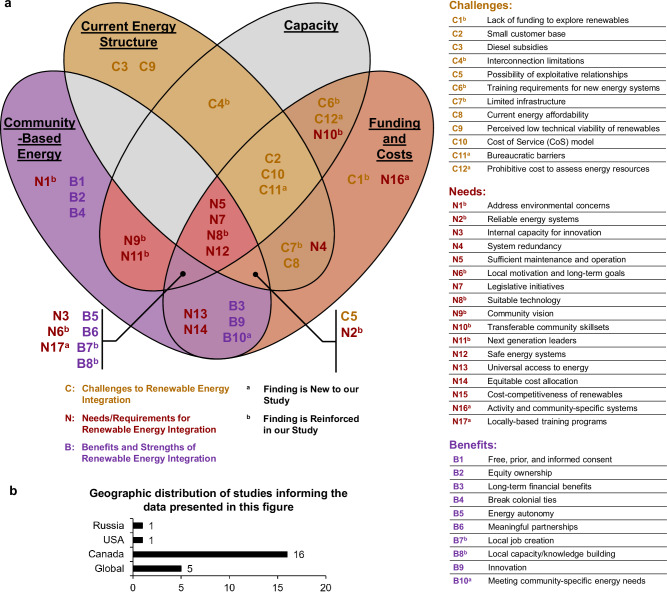
Barriers, needs, and benefits of renewable energy integration for Arctic communities. **a** This heat map four-way Venn diagram illustrates the complexity and interconnectivity of challenges that Arctic communities face to explore and implement renewable energy options, the potential solutions to these challenges, the required framework for projects to be successful, and the benefits of RE projects to Arctic communities. Items with ^b^have been reinforced by our study, and items with ^a^are new findings from our study. These findings were collected as a part of this study as well as from these resources^2,4,10,14–18,26,43–50,95^. This figure is not exclusive, and other factors that impact RE projects in the Arctic may not be included here, especially considering the possible bias towards Canada-based findings as shown in **b** in this figure.

The socio-economic landscape of hydrokinetic and RE development in the Arctic is shaped by community motivations, institutional constraints, and capacity limitations. Indigenous communities in the Arctic are often driven by the desire for energy autonomy, resilience, reduced reliance on diesel, and cultural and environmental preservation. However, these motivations vary across communities, making universal solutions ineffective^43^. Unstructured and semi-structured events were conducted to collect data regarding socioeconomic barriers to RE transition in the global Arctic. This data was used to identify the emerging patterns and thematic categories for the barriers, requirements, and benefits of RE transition shown in Fig. 7. More information on these unstructured and semi-structured methods are available in the “Methods” section.

Figure 7a highlights the interconnected and complex challenges Arctic communities face in adopting RE, as well as the multifaceted conditions needed for success. Beyond cost savings and environmental benefits, RE can also enhance the social and cultural well-being of northern communities. This study and related literature suggest that four primary factors influence Arctic RE projects: funding and costs, capacity, the existing energy structure, and community-based energy. The heat map in Fig. 7a illustrates how the current energy structure, costs, and capacity can hinder progress, but also how community-based initiatives can be leveraged alongside these elements to overcome barriers and advance RE adoption in Arctic communities.

Funding-related barriers and current energy systems are tightly related, which include high capital costs, limited financial support access, administrative delays, little to no financing opportunities, and structural disincentives under current utility revenue models^10,15^. For instance, the cost of service (CoS) model in the Canadian North incentivizes consumption and asset ownership, discouraging efficiency and partnerships with independent RE producers^44^. Even within this model, comparing RE to diesel is difficult because the true cost of heavily subsidized diesel in the Arctic remains uncertain. Until 2023, Nunavut’s legislative framework also prohibited independent power producers, which excluded new entrants from the energy landscape. Communities face further competition for scarce funding resources^45^, while lengthy bureaucratic processes delay project approvals. The high cost of feasibility studies required for funding proposals creates additional barriers. Even when projects move forward, diverse instruments, such as financial support, feed-in-tariffs, grid services, and fiscal incentives, are essential for enabling RE development^45,46^.

Limited capacity can be a major barrier to RE development in the Arctic as well. The current socio-economic disparities present in some Arctic communities, like lower incomes and high food insecurity in Inuit Nunangat^47^, and limited access to proper equipment and skilled labor, limit capacity for RE development^10^. Successful RE implementation requires socio-technical capacity in all project stages supported by seven pillars: local leadership, inter-community networks, long-term energy vision, value creation, energy literacy, transferable skills, and training opportunities^48^. This study emphasizes the importance of having a local, engaged community contact to push project progress. Relationship building and knowledge sharing were pivotal to project success. Furthermore, training local residents has a great impact on project sustainability^26^ and facilitates job creation, a desire revealed in community interviews^49^.

Community-based energy systems are crucial for RE adoption in the Arctic. These systems involve understanding local capacity, local energy needs, social dynamics, and the long-term impacts of energy transitions^48^. Effective approaches include institutional reforms that promote free, prior, and informed consent^19^, equity ownership^50^ or other financial benefit sharing, and local education and engagement^4^. Addressing common concerns, such as project ownership, functionality, funding, logistics, and environmental impacts, is essential^16–18^. In the case of HKE, interviewees raised specific concerns about fish health, caribou migration, biodiversity, and ice-related impacts on turbines^51^. Community-specific energy demand must be considered, as large grid-based systems may not suit small, dispersed Arctic settlements. Instead, micro-grids are often more appropriate^37^. In the Canadian Arctic, where widely spaced cabin-based dwellings support hunting and fishing, modular battery-based systems were identified as better suited to these local needs than large-scale grid integration.

Despite these challenges, many communities show readiness for RE adoption through existing strengths like local leadership, transferable skills, and emerging youth leaders^48^, and communities can still see benefits with externally initiated projects that bring new resources into the community^4^.

## Discussion

HKE can offer a viable RE alternative for remote Arctic communities that are heavily reliant on diesel fuel. Diesel reliance in Arctic communities poses environmental, economic, and logistical challenges, including high fuel costs, supply chain vulnerabilities, infrastructure deficits, and accelerated local climate impacts from black carbon emissions. The Iqaluit Kuunga River (Nunavut, Canada) case study demonstrated technical feasibility in one of the coldest Arctic regions, with the calculated energy yield comparable to an existing installation in Alaska that has been operating for 2 years and offsets community diesel usage by 60–90%.

Upon expanding the assessment nationally and globally, many communities were identified in the Arctic and sub-Arctic showing technical potential for HKE deployment, with 50 communities identified in Canada and 325 communities identified globally. A total theoretical maximum AEP of 1770 MW h year^−1^ m^−2^ was calculated for Canada, and the total global theoretical AEP was 4626 MW h year^−1^ m^−2^ for all identified communities. Diesel offset was calculated for feasible Canadian communities, with ten communities capable of 20% or more reduction in diesel with one turbine installation, and fifteen communities capable of 5% or more reduction with one turbine. Russia exhibited the highest total AEP (2470 MW h year^−1^ m^−2^) but a low average AEP per community (12.5 MW h year^−1^ m^−2^), while Iceland, Canada, Norway, and Finland had higher per-community average AEP, indicating that localized river characteristics are critical for favorable HKE conditions. Favorable HKE sites were typically associated with distinct topographic features, such as bedrock constrictions and steep channel gradients.

The validation of the global Arctic HKE estimates at six geographically diverse river sites revealed that the global river HKE dataset, similar to the CRHE database, is good at identifying feasible communities for further HKE resource investigation. The use cases for these estimates include the identification of feasible communities, the examination of the proximity of communities to river sites with favorable resource potential, and community-to-community resource comparison for energy decision-making. The average difference between modeled AEP and estimates derived from the global dataset was 30.5% (ranging from 4.5 to 53.3%), indicating that the dataset provides a good order-of-magnitude estimate rather than a precise quantification of HKE potential. Given that the required field data collection to follow the IEC^52^ standards of river HKE estimation (see “Methods”) is costly, and that the required hydrodynamic modeling can be time-intensive, such preliminary estimates are valuable for guiding these subsequent, resource-intensive analyses. Overall, the global Arctic HKE dataset provides a strong foundation for targeted HKE investigation and development.

There is a growing body of literature examining the potential impacts of riverine HKE development on local hydrodynamics and sediment transport, and the resulting implications for aquatic ecosystems and wildlife. In general, turbines alter local flow conditions and sediment transport processes, although the magnitude and spatial extent of these effects depend on several key parameters. These parameters include turbine type and size, often represented by tip speed ratio (TSR), where higher TSR typically results in a more localized wake and faster recovery^53,54^; ambient turbulence and flow velocity, with higher turbulence promoting faster wake recovery^55,56^; and the presence of debris, which can increase turbulence near the bed, strengthen scour, and accelerate wake recovery^57^. Turbine design modifications can also influence wake structure. For example, turbine winglets may reduce near-wake vortices while strengthening far-wake vortices^58^. Additional factors include rotor proximity to the bed, which can intensify localized scour^59^, and flow depth, where deeper flows tend to allow faster velocity recovery but slower recovery of turbulence intensity^60^. Sediment grain size appears to have little influence on wake recovery^53^. Reported turbine wake recovery distances generally range from approximately 4D to more than 15D downstream^53–55,57–59,61^, highlighting the varied influence these parameters have on the extent of the turbine’s hydrodynamic impact. Increased turbulence intensity within the wake can lead to localized scour and downstream deposition, linking hydrodynamic changes directly to sediment transport processes. Conversely, sediment scour can further influence hydrodynamics by increasing turbulence and mixing, which may accelerate wake recovery^53^.

Because hydrokinetic turbines are known to generate localized scour, the immediate hyporheic zone may be altered, and this may increase hyporheic exchange depending on the depth of scour^62^. The hyporheic zone provides habitat for diverse assemblages of macroinvertebrates, meiofauna, microorganisms, and various life stages of aquatic organisms^63^. These environments support interconnected chemical, physical, and biological processes that play an important role in river ecosystem functioning^64^. As a result, balancing efficient turbine operation, optimized turbine staging, and protection of river ecology is important. Potential strategies include maintaining sufficient distance between the rotor and the riverbed, optimizing the TSR, and designing turbines to minimize debris accumulation. Despite these localized effects, sediment transport impacts associated with hydrokinetic turbines are considerably smaller than those caused by dam-based hydropower^20,21^. Consequently, hydrokinetic turbines are generally regarded as a relatively environmentally friendly RE technology^65^.

Because turbines also produce measurable changes in local hydrodynamics, it is important to consider their potential influence on fish behavior. Current literature generally indicates that hydrokinetic turbines pose relatively low risk to fish movement, as fish passage was not significantly affected and avoidance behavior was commonly observed across a variety of conditions^66^. Field observations from a turbine installation in the Kvichak River near Igiugig, Alaska, monitored Sockeye Salmon smolt interactions over a 20-day period and found that blade strike events were rare, although some smolts exhibited temporary disorientation due to turbine-induced hydraulic effects^67^. These findings suggest that while impacts appear limited, additional research is needed to fully understand hydrokinetic turbine interactions with fish. Design guidelines have been proposed to further reduce ecological risk by limiting factors, such as physical strike, shear, pressure changes, and excessive turbulence. These guidelines include, but are not limited to, operating turbines at low rotational speeds (around 20 rotations per minute), maintaining low blade tip velocities (below 5 m s^−1^), and maximizing spacing between multiple turbines^65^.

Despite current socioeconomic barriers to RE development in the Arctic—including funding limitations, utility model disincentives, and capacity shortfalls—community-based energy systems, local leadership, and skill development are key enablers for successful renewable transitions. Community-based energy systems can benefit local communities beyond environmental impacts and cost-savings, and HKE shows promise as a community-based RE alternative due to its minimal infrastructure footprint, low environmental impact, and relative ease of installation and maintenance compared to conventional hydropower.

Because the data related to RE socioeconomic factors in northern communities was collected mostly at Canadian locations, a heavy Canadian bias is likely present, specifically in the socioeconomic analysis of this study. Additionally, interviews are more commonly performed in the Western Arctic to gather socio-economic information and to understand the knowledge, values, beliefs, and decision-making processes of stakeholders^68^. As such, the literature examining RE trends in the Arctic may also exhibit a Western bias (see Fig. 7b). Future work could focus on the examination of Global RE socioeconomic factors to reduce such biases. Possible improvements to the approach used in this study include the implementation of interview coding.

## Methods

### General approach

This study employed a four-tiered methodology to evaluate the feasibility of HKE extraction in remote Arctic communities. The first tier involved a site-specific assessment of the HKE resource on a representative Arctic river—the Iqaluit Kuunga River (English name: Sylvia Grinnell River) near Iqaluit, Nunavut, Canada. This included in-situ data collection and subsequent hydrodynamic modeling to estimate the theoretical HKE potential. The second tier focused on identifying remote and rural communities within Canada with favorable conditions for HKE deployment. This national-scale assessment was conducted independently of the global analysis, as the data available for river HKE and remote communities in Canada are more detailed than its global counterparts. The third tier involved a global assessment of riverine HKE resources across the Arctic and sub-Arctic, with the objective of identifying additional rural and remote communities potentially suitable for HKE implementation. The fourth tier of the assessment focused on the evaluation of socioeconomic readiness and community-level capacity for HKE adoption, considering institutional, financial, and logistical factors critical to RE transition in isolated Arctic regions. This final tier aimed to contextualize the technical findings, addressing the practical enablers and barriers to HKE deployment beyond resource availability alone.

### Field data collection at the candidate river site

The case study of HKE resources in a northern river was conducted on the Iqaluit Kuunga River near Iqaluit, Nunavut, Canada in accordance with the International Electrotechnical Commission’s (IEC) standards for river HKE resource estimation and current methods^52,69^. The daily average temperature is −8.6 °C, and the mean annual precipitation is 361.2 mm, comprising 216.6 mm rainfall and 190.0 cm snowfall^28^. The river’s hydrology is primarily driven by snow and ice melt, resulting in a short high-flow period that typically occurs from late June to early July. Two surveys were performed on July 9, 2023, and August 10, 2023, to characterize a high flow (203 m^3^ s^−1^) and low flow (33 m^3^ s^−1^) event, respectively, using a four-beam acoustic Doppler current profiler (ADCP; SonTek M9). The ADCP was deployed using a remote-controlled boat (Oceanscience Q-Boat). During each survey, six transects at a uniform cross section were taken for discharge measurements, three-dimensional velocity profiles were collected in a zigzag pattern with approximate 1/5 river width spacing, and stationary velocity data were collected at the location of highest velocity where the boat could be sufficiently piloted in a stationary position, designated as the OTL (Fig. 1). The sampling frequency was 1 Hz.

The upstream boundary of the survey was determined by the maximum speed that the boat was able to maintain in the rapids without being overtopped. Bathymetry data (riverbed elevation) and water surface elevation data were also collected from the ADCP surveys. The collected ADCP data were geospatially corrected with real-time kinematic differential global positioning system (RTK-DGPS). Due to the high-latitude field conditions and limited satellite availability, the GPS receiver’s azimuth mask angle was reduced from 15° to 10° to increase the number of visible satellites on the horizon in order to employ RTK-DGPS. Research permissions for data collection were granted by the Nunavut Research Institute and the Amarok Hunters and Trappers Association in Iqaluit, Nunavut.

### Numerical hydrodynamic modeling hydrokinetic energy

Hydrodynamic modeling was performed using TELEMAC-2D and TELEMAC-3D, open-source finite element-based numerical hydrodynamic modeling software that solves the shallow water equations and the Navier–Stokes equations, respectively, for simulating free-surface flow^70^. The computational domain was discretized using an unstructured triangular mesh with a uniform node spacing of 2 m, generated in Blue Kenue®^71^. The shoreline topography was derived from the CanElevation Series High Resolution Digital Elevation Model^72^, which provides surface elevations in 1 m resolution.

Model calibration was performed by adjusting the bed roughness (represented with the Strickler coefficient), the velocity diffusivity (via the eddy viscosity), and the upstream bathymetry elevation to minimize the root mean squared error (RMSE) and mean absolute percent error (MAPE) between simulated velocity fields and in situ ADCP measurements for both measured flow conditions. The bed roughness and eddy viscosity were iteratively adjusted following sensitivity analysis, resulting in optimal calibrated values of 30 m^1/3^ s^−1^ and 0.05 m^2^ s^−1^, respectively. To facilitate accurate simulated flow direction and magnitude into the OTL, the upstream bathymetry was calibrated using the points illustrated in Fig. 1. This refinement ensured convergence between modeled and observed flow characteristics within the turbine deployment zone.

To calculate the HKE resource in a typical year of turbine operation, the IEC guidelines for river HKE resource assessment^73^ were followed. For flow characterization, FDCs were generated from the Water Survey of Canada daily hydrometric data from gauge 10UH001 for years 1971–2021 (Fig. 2b). The impact of ice conditions on the theoretical HKE amount was incorporated by defining two distinct operational scenarios: a short-season year characterized by delayed ice break-up and early freeze-up, and a long-season year with early ice break-up and late freeze-up. Two separate FDCs were generated for each condition as maximum and minimum resource estimates. The 51 years of hydrometric gauge measurements were therefore separated into long- and short-season categories. Twelve years of data (1987–1988, 1991, 1997–1998, 2000, 2001–2005, and 2016) were removed from the analysis due to missing or incomplete data. The remaining 40 years were categorized into 25 short-season years (1971–1972, 1974, 1976–1984, 1986, 1989–1990, 1992, 1996, 1999, 2007, 2013, 2015, 2017–2018, and 2020–2021) with more ice-covered days and 14 long-season years (1973, 1975, 1985, 1993–1995, 2006, 2008–2012, 2014, and 2019) with fewer ice-covered days, and separate FDCs were generated for each subset to represent minimum and maximum theoretical HKE availability, respectively. To validate the seasonal classification and the accuracy of Water Survey of Canada ice condition labels (i.e., “ice-covered” measurement labels), visual confirmations of ice break-up and formation timing were performed with Sentinel-2 Level 2A multispectral satellite imagery for the years 2016–2024.

Ten representative flow conditions from each FDC were chosen to represent the entire distribution of flow exceedance probabilities (Fig. 2b). For each selected discharge, velocity flow fields were generated using the previously calibrated and validated TELEMAC hydrodynamic model (Fig. 2c and d). These simulations yielded corresponding (VDCs) (Fig. 2g) at the OTL. The mean stream-wise, depth-averaged velocity at the OTL was used to calculate the power using Eq. 1, resulting in the generation of (PDCs) (Fig. 2h). The PDCs were interpolated using Eq. 2 to calculate the AEP.

1 $$P=\frac{1}{2}{A\rho v}^{3}$$

2 $${AEP}={N}_{h}\mathop{\sum }\limits_{i=1}^{{N}_{b}}{P}_{i}{B}_{i}$$

where *P* is the hydrokinetic power (W), *A* is the turbine cross-sectional area (assumed to be 1 m^2^ for standardization), *ρ* is the density of water (kg m^−3^), *v* is the flow velocity (m s^−1^), AEP is the annual energy production (MW h year^−1^ m^−2^), *N*_*h*_ is the number of turbine operation hours in a year (hours), *i* is the number of points on the PDC, *P*_*i*_ is the power at a particular flow exceedance (W), and *B*_*i*_ is the width on the PDC that the point represents as a decimal probability.

Because the Iqaluit Kuunga River freezes to the riverbed in the winter at the investigated location, the hydrokinetic turbine would need to be removed prior to freeze-up in the fall and reinstalled post ice break-up in the spring (mean season length of 103 days). This seasonal deployment schedule was explicitly integrated into the AEP estimation through the method used to construct the seasonal FDCs, ensuring that resource availability reflects realistic operational windows.

### Technical in-stream hydrokinetic potential for remote Canadian communities

To identify remote Arctic and sub-Arctic communities in Canada with potential for HKE deployment, two national geospatial datasets were utilized: the CRHE database^35^ and the Remote Community Energy (RCE) database^36^. The CRHE database includes estimates of HKE for all rivers in Canada that can exceed 100 m^3^ s^−1^ at any point in their hydrological cycle and are greater than 20 m in width, and has been comprehensively validated with rivers of similar characteristics.

The river HKE estimates in the CRHE were derived using a multi-step methodology. Discharge estimates were extracted from two datasets. The first dataset consisted of modeled flows in southern Canada obtained by multiple linear regression and canonical correlation analysis informed by Water Survey of Canada flow records to group statistically similar hydrometric data^74^. The approach was calibrated by adjusting the chi-squared distance value (alpha) to minimize the average of RMSE flow values. These flows were validated using the jackknife or leave-one-out approach and showed good agreement with measured values (*R*^2^ = 0.95). The second river flow dataset consisted of modeled flows in northern Canada obtained by downscaling hydrological data and converting runoff estimates into streamflow via the WATROUTE routing scheme^75^. The data was validated visually by comparing hydrographs of six hydrometric gauge locations, showing good agreement^76^.

River depth was estimated from hydraulic geometry relationships^74^: power law-based relationships between river depth and river flow that were derived at Water Survey of Canada hydrometric gauge stations^74,77^. These hydraulic geometry relationships were created for unregulated channels^78,79^ based on the prediction of bank-full depth from a design flow as originally developed by Leopold and Maddock^80^. They were validated using a jackknife (or leave-one-out) approach for each hydrometric station and showed good agreement in regions in steep or bedrock-based channels (*R*^2^ = 0.73, 0.76) and poorer agreement in regions with more gradual gradients and complex hydrology (*R*^2^ = 0.57, 0.30).

River width was extracted from Sentinel-2 multispectral satellite imagery^35^. The method used to create surface water maps was the automated water extraction index, no shadow, and the river widths were measured using an automated GIS model^35^. This remote sensing-based approach to measuring river widths was calibrated extensively using four machine learning approaches and five spectral index approaches in four geographically diverse locations in two different seasonal conditions^81,82^. Calibration was also performed using sensitivity analysis of thresholds. The calibration process for river width measurements for the CRHE database is described fully in Kirby et al.^81^. Validation was performed at 35 geographically diverse river locations in different flow conditions (a total of 156 measurements used for validation), resulting in an *R*^2^ of 0.95^35^.

A proximity analysis was performed between river cross sections exceeding a theoretical HKE density of 10 MW h year^−1^ m^−2^ and the 178 communities in the RCE database. Communities within a 10 km radius of these cross sections were flagged as candidates for HKE integration. Using community-level data from the RCE database, including annual energy consumption (in MWh per year) and primary electricity generation source, the potential diesel offset achievable by a single hydrokinetic turbine was estimated for each community. Furthermore, a statistical correlation analysis using the coefficient of determination (*R*^2^) was conducted to evaluate the relationship between mean annual air temperature and community energy consumption, with the objective of determining whether colder communities, which typically exhibit higher heating-related electricity demand, correlate with lower diesel offset potential. This analysis aimed to assess how climatic factors might influence the technical and economic feasibility of HKE adoption in northern remote communities.

### Calculation of technical in-stream hydrokinetic potential for global Arctic communities

To identify communities feasible for river HKE deployment in the global Arctic, estimates of HKE were derived using Eqs. 1 and 2 for all rivers above 55°N. Community locations were extracted from the Geographic Names Server (GNS), a geospatial database maintained by the National Geospatial-Intelligence Agency that contains standardized place names approved by the United States Board on Geographic Names^83^. A proximity analysis was performed between community locations and river cross sections with an estimated HKE exceeding 10 MW h year^−1^ m^−2^. Communities located within 10 km of a favorable river location were identified as candidates for HKE deployment. Each of the inputs to the estimation of river HKE (river flow, depth, and width) were calibrated separately in the works that they were acquired. The derived estimates of river HKE were validated at six separate river sites: three Canadian sites in which full HKE resource estimations were performed according to the IEC^52^ standards by the authors, and three other independent, international sites where HKE resources were estimated. The three independent river sites were not necessarily performed according to the IEC^52^ standards, yet they still provide a useful metric for comparison to the globally derived HKE estimates.

The global Arctic HKE estimates were derived using a methodology consistent with the IEC^52^ standards for river HKE resource estimation, wherein flow, velocity, and power duration curves were developed to compute AEP for each river cross section. This is a similar methodology that was applied to the Iqaluit Kuunga River case study. To generate the FDCs, the Global Reach-level a priori Discharge Estimates (GRADES) dataset^84^ was used, which contains model-derived daily discharge estimates for 1980–2013 at a global scale. The GRADES dataset was calibrated using streamflow characteristics rather than parameter regionalization, resulting in improved model performance even at ungauged locations^85^. Model parameters were calibrated and bias corrected against a machine learning-derived map describing global runoff characteristics with discharge observations from 3000 to 4000 naturalized catchments^86^. The results were validated at over 14,000 hydrometric gauges with more than 3 years of data. This comparison demonstrated the robustness and high accuracy of the derived dataset, with the majority of the 14,000 gauge locations having a percent bias between ±20%^84^. The FDCs from the GRADES data were spatially coupled to river locations using MERIT hydro streamlines^87^.

River channel geometry was estimated from two sources: river depth from the same hydraulic geometry relationships used in the CRHE database^35,74^ and river width from the Global River Widths from Landsat (GRWL) dataset^88^. The GRWL dataset includes estimates of river width at mean annual flow with an average spatial resolution of 35 m. This dataset was derived from Landsat TM, ETM+, and OLI scenes acquired at approximated months of mean discharge conditions^88^. The modified normalized difference water index was used with a dynamic thresholding procedure^89^ to create surface water maps, and river centerlines and widths were extracted using the RivWidth algorithm^90^. Visual inspection was used to calibrate the threshold and correct for any gaps, while validation was performed at 1250 United States Geological Survey stream gauge locations, resulting in a RMSE of 25.2 m^88^.

For the calculation of velocity and HKE, a rectangular channel shape was assumed. VDCs were derived for each cross-section by estimating the cross-sectional mean velocity for each FDC exceedance probability by conservation of mass, using the derived river depth at each flow exceedance and a constant river width at mean annual flow. Because multiple width measurements across different flow conditions are not available globally in the GRWL dataset, as they are in the CRHE database, estimates of velocity at high exceedance and low exceedance probabilities on the VDC are likely to contain some error. The power at each exceedance was calculated using Eq. 1, assuming a turbine cross-sectional area of 1 m² for standardization across sites. The resulting PDCs were interpolated using Eq. 2 to compute the AEP at each river cross section.

The global Arctic HKE estimates were validated at locations where measurements of river HKE had been previously derived using a combination of field measurements (usually using ADCP) and numerical modeling. Three validation locations were provided by the authors, and three additional validation locations were acquired from independent literature. The three Canadian validation river sites provided by the authors were the Iqaluit Kuunga River (63.787°N, −68.622°W), the Riviere-Rouge (45.645°N, −74.691°W)^69^, and the Spanish River (46.177°N, −82.091°W)^91^. Further details on these validation locations is available in ref. ^35^.

The three independent, international validation locations were the Tanana River near Nenana, Alaska, United States of America (64.568°N, −149.076°W)^92^, the Kvichak River near Igiugig, Alaska, United States of America (59.324°N, −155.915°W)^26^, and the Neris River near Jonava, Lithuania (55.0687°N, 24.282°E)^93^. The HKE study on the Tanana River reported 4500 W m^−2^ for the average power in the area of maximum energy in late August when field measurements were taken^92^. Converting to comparable values by using monthly AEP values, as power in the Tanana River wasn’t reported as AEP and rather as power density, an estimate of 3.87 MW h year^−1^ m^−2^ was derived from our study at this river location, while the model calibrated with ADCP measurements reported 4.84 MW h year^−1^ m^−2^^92^. The HKE study on the Kvichak River reported the annual power output, depending on the siting of the device, to be between 150.28 and 222.59 MW h^26^. Converting to comparable values, using a 9 m^2^ capture area for one ORPC RivGen turbine, the modeled energy in the Kvichak River was 24.7 MW h year^−1^ m^−2^. The global HKE database developed in this work estimated the hydrokinetic resource to be 23.58 MW h year^−1^ m^−2^. The HKE study on the Neris River reported a mean power density of 0.24 kW m^−2^ at Jonava gauge station in a normal year^93^. Converting to comparable values, the AEP for this river location is 2.10 MW h year^−1^ m^−2^. The global HKE database developed in this work estimated the hydrokinetic resource to be 3.22 MW h year^−1^ m^−2^.

### Socioeconomic status of renewable energy transition in the global Arctic

Feedback on river HKE development and broader RE transition efforts in Arctic regions was collected through in situ community engagement during fieldwork near Iqaluit, Nunavut, Canada, and via semi-structured knowledge dissemination events involving local residents and key informants. Ethical clearance to perform this work was granted by the Nunavut Research Institute (license number 02 032 23N-M, approved June 6, 2023), the Nunavut Impact Review Board (file number 23YN010, application number 125783, approved May 25, 2023), and the Amaruq Hunters and Trappers Association (approved June 30, 2023, by the vice-chairperson of the Amaruq HTA). Proper ethical clearance was only possible through local community member participation via collaborators at Polar Outfitting in Iqaluit, Nunavut. The authors gathered data regarding barriers to RE transitions in northern communities, the general interest in RE systems, and if the participants expected any benefits from RE in their communities. The participants were also asked if there were any foreseeable challenges to HKE deployment in their communities. Participants were asked open- and closed-ended questions to elicit insights on institutional, financial, technical, and logistical constraints to RE deployment. Table 1 presents the community engagement and structured dissemination events, undertaken from which the socioeconomic data was collected.

**Table 1:** Stakeholder and representative groups from which data on barriers, requirements, and benefits of Arctic renewable energy transition were collected

Event	Group demographic	Date(s)	Approach applied^68^	Sampling approach^68^	Approximate number of respondents
Arctic Circle Assembly 2024; Reykjavik, Iceland	Arctic Circle Assembly participants	October 17, 2024	Structured dissemination and discussion (semi-structured)	Key informant sampling	10
At Nunavut Arctic College; Iqaluit, NU, Canada	Students and instructors at Nunavut Arctic College	October 3, 2024	Structured dissemination and discussion (semi-structured)	Key informant sampling	10
During proposal approval and during informal dissemination; Iqaluit, NU, Canada	Amaruq Hunters and Trappers Association members	October 3, 2024 May 21–26, 2023	Informal (unstructured)	Key informant sampling	5
At SevenGen Summit 2024; Iqaluit, NU, Canada	Canadian Indigenous youth, university age	October 1–3, 2024	Structured dissemination, field trip, workshop, and discussion (semi-structured)	Representative sampling	60
During field work collection; Iqaluit, NU, Canada	Polar Outfitting employees	August 9–12, 2023 July 5–10, 2023	Informal (unstructured)	Representative sampling	5
During field work and community engagement; Iqaluit, NU and Resolute Bay, NU, Canada	Northern community members	August 9–12, 2023 July 5–10, 2023 May 17–26, 2023	Informal (unstructured)	Snowball sampling	10
At Northern Lights 2023 Conference and Trade Show; Ottawa, ON, Canada	Northern Lights Conference participants	February 9–10, 2023	Informal (unstructured)	Random sampling	10
At ArcticNet 2022 Conference; Toronto, ON, Canada	ArcticNet Conference participants	December 4–8, 2022	Structured dissemination and discussion (semi-structured)	Key informant sampling	20

The approach employed in this study was an unstructured and semi-structured approach to information gathering. The rationale for the use of an unstructured and semi-structured approach was based on expert opinion (i.e., professionals with many years of experience working with and in Arctic and remote communities) regarding effective methods for gathering information in northern communities. A formal interview structure originally did not yield strong engagement; one pilot using formal questions was conducted with poor results. As a result, the approach was changed to unstructured and semi-structured interviews, as originally suggested by expert opinion. Informal and semi-structured approaches allow for greater flexibility. This approach also allowed for sensitivity and caution in establishing an ethical relationship between the interviewee and the researcher. Additionally, the dissemination sessions, interviews, and discussions were not transcribed. Rather, generalized points, ideas, and themes were collected instead of specific wording.

To complement the empirical feedback, a comprehensive literature review of RE transitions across the global Arctic was conducted. Both sources of information, primary community input and secondary literature, were categorized into four identified thematic categories: funding and costs, capacity, current energy structures, and community-based energy. Key findings from both the community engagement and literature review were mapped to these categories to identify recurring challenges and enabling conditions. For example, training requirements for new energy systems were interpreted as both a capacity issue and a cost-related barrier, given the requirement for both time investment and financial resources. Categorizing the key findings provided a structured framework for interpreting the multifaceted social and institutional dynamics that shape RE deployment in remote Arctic communities.

### Reporting summary

Further information on research design is available in the Nature Portfolio Reporting Summary linked to this article.

## Supplementary information

Description of Additional Supplementary Files

Supplementary Data 1

Supplementary Data 2

Reporting Summary

Transparent Peer Review File

Supplementary Data 3

## Data Availability

The Iqaluit Kuunga River raw data are available under restricted access for community ownership, and access can be obtained upon request. The Canada remote communities’ hydrokinetic energy and diesel offset data estimates generated in this study are provided in the Supplementary Information/Source data file (Supplementary Data 1, name: Canada_RCED_HKE, file type: point shapefiles in geodatabase). The global Arctic and sub-Arctic remote communities’ hydrokinetic energy estimates generated in this study are provided in the Supplementary Information/Source data file (Supplementary Data 2, name: Global_Arctic_HKE, file type: point shapefiles in geodatabase). The global Arctic and sub-Arctic river hydrokinetic energy estimate data generated in this study are available in the National Research Council (NRC) Digital Repository under the database name global Arctic and sub-Arctic River Hydrokinetic Energy Database [10.4224/40004011] (Supplementary Data 3, name: GHKE_DB, file type: line shapefiles in geodatabase).
