# Supplementary material for: The in-stream renewable energy potential of rivers for remote communities in the global Arctic
Source: Nat Commun. 2026 Jun 16;17:7653. doi: 10.1038/s41467-026-74292-6 (PMC13433784; doi:10.1038/s41467-026-74292-6)
Supplement: Supplementary file 1 [file 41467_2026_74292_MOESM1_ESM.pdf]

Description of Additional Supplementary Files

Supplementary Data 1: The Canada remote communities' hydrokinetic energy and diesel offset data estimates (name: Canada_RCED_HKE, file type: point shapefiles in geodatabase).

Supplementary Data 2: The Global Arctic and sub-Arctic remote communities' hydrokinetic energy estimates (name: Global_Arctic_HKE, file type: point shapefiles in geodatabase).

Supplementary Data 3: The Global Arctic and sub-Arctic River hydrokinetic energy estimate data (name: GHKE_DB, file type: line shapefiles in geodatabase). Available in the National Research Council (NRC) Digital Repository under database name Global Arctic and sub-Arctic River Hydrokinetic Energy Database [https://doi.org/10.4224/40004011].

https://nrc-digital-repository.canada.ca/eng/view/object/?id=c99c30d0-1951-4c07-a319-aa31577b965d
