# Supplementary material for: The in-stream renewable energy potential of rivers for remote communities in the global Arctic
Source: Nat Commun. 2026 Jun 16;17:7653. doi: 10.1038/s41467-026-74292-6 (PMC13433784; doi:10.1038/s41467-026-74292-6)
Supplement: Supplementary file 2 [file 41467_2026_74292_MOESM4_ESM.pdf]

nature portfolio

Corresponding author(s): Katelyn Kirby

Last updated by author(s): Mar 20, 2026

Reporting Summary

Nature Portfolio wishes to improve the reproducibility of the work that we publish. This form provides structure for consistency and transparency in reporting. For further information on Nature Portfolio policies, see our Editorial Policies and the Editorial Policy Checklist.

Statistics

For all statistical analyses, confirm that the following items are present in the figure legend, table legend, main text, or Methods section.

n/a	Confirmed	
☐	☒	The exact sample size (*n*) for each experimental group/condition, given as a discrete number and unit of measurement
☒	☐	A statement on whether measurements were taken from distinct samples or whether the same sample was measured repeatedly
☒	☐	The statistical test(s) used AND whether they are one- or two-sided<br>*Only common tests should be described solely by name; describe more complex techniques in the Methods section.*
☒	☐	A description of all covariates tested
☒	☐	A description of any assumptions or corrections, such as tests of normality and adjustment for multiple comparisons
☐	☒	A full description of the statistical parameters including central tendency (e.g. means) or other basic estimates (e.g. regression coefficient) AND variation (e.g. standard deviation) or associated estimates of uncertainty (e.g. confidence intervals)
☒	☐	For null hypothesis testing, the test statistic (e.g. *F*, *t*, *r*) with confidence intervals, effect sizes, degrees of freedom and *P* value noted<br>*Give P values as exact values whenever suitable.*
☒	☐	For Bayesian analysis, information on the choice of priors and Markov chain Monte Carlo settings
☒	☐	For hierarchical and complex designs, identification of the appropriate level for tests and full reporting of outcomes
☒	☐	Estimates of effect sizes (e.g. Cohen's *d*, Pearson's *r*), indicating how they were calculated

Our web collection on statistics for biologists contains articles on many of the points above.

Software and code

Policy information about availability of computer code

Data collection	RiverSurveyor Live Version 4.3 was used to collect and process the ADCP field data. The ADCP used in the field data collection was the SonTek RiverSurvey model M9.
Data analysis	No custom code was developed specifically for this study. A pre-existing, general-purpose Matlab Version R2023 code (not central to the research) was used to clean and process the ADCP field data. This code is available upon request. ArcGIS Pro Version 3.5.4 was used to analyze and process the geospatial data.

For manuscripts utilizing custom algorithms or software that are central to the research but not yet described in published literature, software must be made available to editors and reviewers. We strongly encourage code deposition in a community repository (e.g. GitHub). See the Nature Portfolio guidelines for submitting code & software for further information.

Data

Policy information about availability of data

All manuscripts must include a data availability statement. This statement should provide the following information, where applicable:

- Accession codes, unique identifiers, or web links for publicly available datasets
- A description of any restrictions on data availability
- For clinical datasets or third party data, please ensure that the statement adheres to our policy

The following datasets are included in the supplementary material:

(1) Canada remote communities' hydrokinetic energy and diesel offset estimates, point shapefiles in geodatabase (name: Canada_RCED_HKE)
(2) Global Arctic and sub-Arctic remote communities' hydrokinetic energy estimates, point shapefiles in geodatabase (name: Global_Arctic_HKE)
The Global Arctic and sub-Arctic river hydrokinetic energy estimates, line shapefiles in geodatabase (name: GHKE_DB), are available through the National Research Council of Canada data repository.
All other data used in and generated from this analysis are available upon request, due to community data ownership.

Research involving human participants, their data, or biological material

Policy information about studies with human participants or human data. See also policy information about sex, gender (identity/presentation), and sexual orientation and race, ethnicity and racism.

Reporting on sex and gender	Sex and gender were not considered in the study design.
Reporting on race, ethnicity, or other socially relevant groupings	Race, ethnicity, or other socially relevant groupings were not considered in the study design.
Population characteristics	Population characteristics were not considered in the study design.
Recruitment	Participants for the "socioeconomic status of renewable energy transition in the global Arctic" were recruited through in-situ community engagement during fieldwork near Iqaluit, Nunavut, Canada, and via semi-structured knowledge dissemination events involving local residents and key informants. This semi-structured and unstructured approach collected empirical feedback from participants only residing in northern Canada. This introduced a spatial bias into the results. However, this was attempted to be offset through the addition of secondary literature of the same topic collected in other locations. Limitations to this approach are discussed in the manuscript.<br><br>Informed consent was obtained from all participants involved in community engagement and knowledge dissemination activities prior to participation. Participants were informed of the purpose of the study, the voluntary nature of their involvement, and that their input would be used in aggregated, non-identifiable form. No personally identifiable information was collected.
Ethics oversight	The study protocol was approved by the Nunavut Research Institute (Iqaluit, NU, Canada), the Research Ethics Board of the University of Ottawa (Ottawa, ON, Canada), and the Amaruq Hunters and Trappers Association (Iqaluit, NU, Canada).

Note that full information on the approval of the study protocol must also be provided in the manuscript.

Field-specific reporting

Please select the one below that is the best fit for your research. If you are not sure, read the appropriate sections before making your selection.

☐ Life sciences ☐ Behavioural & social sciences ☒ Ecological, evolutionary & environmental sciences

For a reference copy of the document with all sections, see nature.com/documents/nr-reporting-summary-flat.pdf

Ecological, evolutionary & environmental sciences study design

All studies must disclose on these points even when the disclosure is negative.

Study description	This study evaluates HKE technical and socioeconomic feasibility through (i) a detailed case study of the Iqaluit Kuunga River in Nunavut, Canada, (ii) a global analysis to identify feasible communities, and (iii) an examination of Arctic socioeconomic readiness for energy transition.<br><br>The study employs a multi-tiered, mixed-methods design combining quantitative modelling, geospatial analysis, and qualitative socioeconomic assessment rather than a controlled experimental design with treatments. This is an observational, model-based, multi-scale study with no controlled experimental treatments, relying on spatiotemporal replication, scenario analysis, and integrated quantitative-qualitative synthesis to evaluate hydrokinetic energy feasibility.<br><br>The study design structure employs a hierarchical and sequential approach (site-scale to national-scale to global-scale) with elements of scenario-based modelling rather than factorial experimentation.
Research sample	The research sample for part (i) of the study was the Iqaluit Kuunga River, a representative Arctic river located near a northern community in Canada. This river was chosen due to its flow characteristics and local geomorphology indicating that it was a promising candidate for hydrokinetic energy development. The research sample for part (ii) of the study focused on the global Arctic due to the presence of small, remote and isolated communities currently reliant on diesel for heating and electricity. These communities would most benefit from community-based, decentralized power sources. Data for this portion of the study was derived from the Canadian River Hydrokinetic Energy (CRHE) database, the Remote Community Energy Database (RCED), the Geographic Names Server (GNS), the Global Reach-level a priori Discharge Estimates (GRADES) dataset, and the Global River Widths from Landsat (GRWL) dataset. The research sample for part (iii) of the study was the global Arctic to provide nuance and practical applicability to the technical findings of part (ii) of the study.

Sampling strategy	For the field data collection, the river characteristics of the Iqaluit Kuunga River were measured in two different flow conditions. This sampling strategy was employed due to the requirements for calibrating and validating a hydrodynamic model to represent a multitude of flow conditions beyond the two that were measured in field. This allowed for proper characterization of the hydrokinetic energy resource throughout all seasonal conditions.<br><br>At the modelling stage, representative flow conditions were selected using flow duration curves derived from long-term hydrometric records (1971–2021). Ten flow conditions were sampled from each of two seasonal scenarios (short- and long-season), ensuring coverage across the full range of flow exceedance probabilities.<br><br>No formal statistical sample size calculation was performed. Sample sizes were determined based on (i) standard practices for hydrodynamic field data collection and model calibration, (ii) the need to capture representative hydrological variability through flow duration curve sampling, and (iii) the use of complete, publicly available datasets at national and global scales. These approaches are widely accepted in hydrological and resource assessment studies and are sufficient to ensure robust estimation of hydrokinetic energy potential, as per the International Electrotechnical Commission (2019) guidelines for hydrokinetic energy estimation.
Data collection	Field data were collected using an acoustic Doppler current profiler (ADCP) mounted on a remote-controlled vessel (Oceanscience Q-Boat) and was performed via collaboration between University of Ottawa researchers and Polar Outfitting, a local Inuit-owned and operated outfitting company. Data were collected at the full area of interest in the river, as well as upstream and downstream of the area of interest, to ensure accurate model calibration and validation for the river.
Timing and spatial scale	For part (i) of the study, data were collected during two different flow conditions. The dates of the data collection were planned based on insights from the study site's hydrograph based on hydrometric gauge station data. This was done to ensure that two flows of different magnitudes were measured. The first data sampling was performed on July 9, 2023 during a high flow condition. the second data sampling was performed on August 10, 2023 during a low flow condition. Each data collection campaign lasted approximately 3 hours to collect data across river area of interest. The ADCP data collection frequency was set to 1 Hz. The spatial extent of data collection in the river was 10 meters upstream of the area of interest (i.e., area with the highest flow velocity) and 200 meters downstream of the area of interest to ensure enough data were collected to accurately calibrate the hydrodynamic model. Data could not be collected more than 10 meters upstream of the area of interest due to unsafe river conditions (i.e., rapids).
Data exclusions	No data were excluded from the study.
Reproducibility	The site-specific hydrodynamic modelling completed in part (i) of this study was calibrated and validated using independent field measurements collected under different flow conditions, demonstrating consistent model performance across scenarios. Representative flow conditions were systematically selected from long-term hydrometric records to ensure that results were not dependent on a single observation period. Field measurements were conducted during two independent sampling campaigns (July and August 2023), representing high- and low-flow conditions, respectively. Within each campaign, multiple repeated transects (six per cross-section) were performed to ensure reliable discharge estimation. Hydrodynamic simulations were conducted across multiple independently defined flow conditions derived from long-term hydrometric records (1971–2021), including ten representative flows for each of two seasonal scenarios. These simulations collectively capture a wide range of hydrological conditions rather than relying on a single experiment or data collection campaign.<br><br>At larger scales completed in part (ii) of this study, hydrokinetic energy estimates derived from national and global datasets were validated against multiple independent river sites, showing good agreement and consistent order-of-magnitude accuracy. These analyses were performed using complete datasets and are therefore not based on experimental replication in the traditional sense.
Randomization	Randomization is not applicable to this study because the research design is observational and process-based rather than experimental. The analysis relies on field measurements, hydrodynamic modelling, and large-scale geospatial datasets to characterize existing river conditions and estimate hydrokinetic energy potential, without manipulating variables or assigning treatments to controlled groups.
Blinding	Blinding was not applied in this study, as it was not relevant to the research design. The study is based on objective field measurements (e.g., ADCP-derived velocity, bathymetry, and water surface elevation), numerical hydrodynamic modelling, and analysis of established geospatial and hydrological datasets. These data sources are instrument-driven and quantitative, minimizing the potential for subjective bias during data acquisition and analysis. Additionally, the modelling workflows for both parts (i) and (ii) of this study (including calibration, validation, and generation of flow, velocity, and power duration curves) follow standardized procedures, where outcomes are governed by physical equations and input data rather than researcher interpretation. For part (iii) of this study, unstructured and semi-structured engagement methods were used to gather general themes and insights rather than responses to predefined hypotheses. As such, blinding was not feasible, nor necessary, given the exploratory and qualitative nature of this portion of the study.

Did the study involve field work? ☒ Yes ☐ No

Field work, collection and transport

Field conditions	Two field data collections were performed. The first on July 9, 2023 was during a high river flow condition. The mean air temperature was 8.6 degrees Celsius with no precipitation. The second on August 10, 2023 was during a low river flow condition. The daytime air temperature was 9.6 degrees Celsius with no precipitation.
Location	Field data was collected in the Iqaluit Kuunga (Sylvia Grinnell) River near Iqaluit, NU, Canada. The latitude of the study location was 63.788 and the longitude of the study location was -68.622.

Access & import/export	The field location was accessed via all-terrain vehicle (ATV) with the field equipment being towed behind in a qamutiik. This approach was proposed by local community members and approved by the Amaruq Hunters and Trappers Association (Iqaluit, NU, Canada) on June 30, 2023.
Disturbance	Disturbance to the site and the route used to access the site were minimized through consultation with the Amaruq Hunters and Trappers Association. Use of the qamutiik to transport field equipment minimized disturbance to Arctic vegetation. Minimizing disturbance to Arctic vegetation was achieved by using established, well-travelled paths. Additionally, field data was collected with a battery-operated remote controlled boat in the Iqaluit Kuunga River to avoid any pollution to the river caused by fossil fuel-driven propellers.

Reporting for specific materials, systems and methods

We require information from authors about some types of materials, experimental systems and methods used in many studies. Here, indicate whether each material, system or method listed is relevant to your study. If you are not sure if a list item applies to your research, read the appropriate section before selecting a response.

Materials & experimental systems

n/a	Involved in the study	
☒	☐	Antibodies
☒	☐	Eukaryotic cell lines
☒	☐	Palaeontology and archaeology
☒	☐	Animals and other organisms
☒	☐	Clinical data
☒	☐	Dual use research of concern
☒	☐	Plants

Methods

n/a	Involved in the study	
☒	☐	ChIP-seq
☒	☐	Flow cytometry
☒	☐	MRI-based neuroimaging

Plants

Seed stocks	No plants were collected as part of this study.
Novel plant genotypes	*Describe the methods by which all novel plant genotypes were produced. This includes those generated by transgenic approaches, gene editing, chemical/radiation-based mutagenesis and hybridization. For transgenic lines, describe the transformation method, the number of independent lines analyzed and the generation upon which experiments were performed. For gene-edited lines, describe the editor used, the endogenous sequence targeted for editing, the targeting guide RNA sequence (if applicable) and how the editor was applied.*
Authentication	*Describe any authentication procedures for each seed stock used or novel genotype generated. Describe any experiments used to assess the effect of a mutation and, where applicable, how potential secondary effects (e.g. second site T-DNA insertions, mosiacism, off-target gene editing) were examined.*
