# Supplementary material for: The in-stream renewable energy potential of rivers for remote communities in the global Arctic
Source: Nat Commun. 2026 Jun 16;17:7653. doi: 10.1038/s41467-026-74292-6 (PMC13433784; doi:10.1038/s41467-026-74292-6)
Supplement: Supplementary file 3 [file 41467_2026_74292_MOESM5_ESM.pdf]

nature portfolio

Peer Review File

The In-Stream Renewable Energy Potential of Rivers for Remote Communities in the Global Arctic

Corresponding Author: Dr Katelyn Kirby

This file contains all reviewer reports in order by version, followed by all author rebuttals in order by version.

Version 0:

Reviewer comments:

Reviewer #1

(Remarks to the Author)
The research under review provides a sound assessment of the feasibility of hydrokinetic energy potential of rivers in remote communities of the Global Artic. The study starts with a detaled case study of the Iqaluit Kuunga River in Nunavut, Canada, then upscales the methodology for Canada and 8 countries / regions in the Global Artic.
The work is original, relevant and has a significant contribution through a thorough analysis of technical and geo-spatial conditions to deploy hydrokinetic energy instalations in rivers, accross remote areas. The significance of the study lays not only in the multi-scale analysis of potentials, but also the outline of chalenges, needs and benefits for the implementation of a local renewable energy grid.
The discussion points and conclusions of the study are supported by results.
I could not identify flaws in the data analysis and interpretation. The methodology is coherent, clear and provides a complete work flow for replicability.
I have no further comments or indications for changing the manuscript's content.

Reviewer #2

(Remarks to the Author)
Overall, the authors present an interesting and timely study of in-stream renewable energy potential for remote communities in the Arctic based on hydrokinetic energy from field collected data and existing datasets for Canadian rivers. Such an analysis can provide valuable insights into energy transitions and resilience for remote communities that often rely on fossil fuel generation. The research evaluated the technical and socioeconomic feasibility of hydrokinetic energy through a single case study, a global analysis, and an examination of readiness for this technology based on socioeconomic status of various remote communities. Below are my suggestions and comments.

Major comments
Methodology: The study leverages a four-tiered approach to evaluate the feasibility of HKE in remote Arctic communities. While the approach is outlined logically, it raises some concerns regarding its robustness. A key limitation to this study is that field collected data at a single site was used to calibrate results for HKE potential across Canada. Because river morphology, flow regimes, and land-use contexts vary widely, it is unclear whether this single-site calibration is transferable to other settings. This approach risks overfitting the model to local conditions rather than demonstrating broad applicability. At minimum, calibration and validation should rely on independent datasets, preferably from different river systems, and the authors should justify why the selected site is representative of the broader population of rivers. A cross-site validation or transferability analysis would also be valuable. If such analyses are not feasible, the manuscript should temper its broader claims accordingly.

Methodology: Feedback on river HKE development and broader renewable energy transition efforts in Arctic regions were collected through in-situ community engagement during field work near Iqaluit, Nunavut Canada and through structured knowledge dissemination events involving local residents. It is not clear from the methods the protocol used to collect such data and the transferability of these results to the broader Arctic community. Please provide additional details regarding questions asked, number of respondents, characteristics of respondents, and literature clarifying the approach used in the context of the study and broader social science methods.

Introduction: Limitations to this study include optimal sizing of HKE projects, reliability of HKE, and policy support. However, there is a broad and growing literature on the potential impacts of renewable energy development on ecosystems and wildlife. Please either include an analysis of the potential impacts HKE could have on water quality, habitat structure, sediment dynamics, and freshwater biotic communities, or include a dedicated discussion of the potential ecological implications of such development.

Minor comments
Line 54 – consider including dollars in parenthesis when reporting international currency's; 3 rubles per kWh (x $ per kWh)
Line 485 – The CRHE dataset contains estimates of KHE for all rivers in Canada exceeding 100 m3/s and are greater than 20 m in width. Do these parameters reflect the in-situ river used for calibration and validation?
Line 37 through 40 – what is the relative risk caused by these changes?
Line 31 through 33 – Does this statistic include European Arctic communities?
When necessary, include text directing the reader to the methods.

Version 1:

Reviewer comments:

Reviewer #2

(Remarks to the Author)
The authors have fully addressed comments raised during my previous review of this manuscript. It is my assessment that the revised work is now suitable for publication.

Open Access This Peer Review File is licensed under a Creative Commons Attribution 4.0 International License, which permits use, sharing, adaptation, distribution and reproduction in any medium or format, as long as you give appropriate credit to the original author(s) and the source, provide a link to the Creative Commons license, and indicate if changes were made.
In cases where reviewers are anonymous, credit should be given to 'Anonymous Referee' and the source.
The images or other third party material in this Peer Review File are included in the article's Creative Commons license, unless indicated otherwise in a credit line to the material. If material is not included in the article's Creative Commons license and your intended use is not permitted by statutory regulation or exceeds the permitted use, you will need to obtain permission directly from the copyright holder.
To view a copy of this license, visit https://creativecommons.org/licenses/by/4.0/

Response to reviewer comments for

Manuscript Ref. No.: NCOMMS-25-88830-T

The In-Stream Renewable Energy Potential of Rivers for Remote Communities in the Global Arctic,

by Katelyn Kirby, Colin D. Rennie, Ioan Nistor, Cameron Johnstone, Stephanie Ordonez Sanchez, Song Fu, Julien Cousineau, Heather Shilton

The authors of this manuscript are thankful to both reviewers for their helpful comments and critiques to improve the content, organization, and message of the manuscript. The comments of the reviewers have been addressed below with care and consideration. The comments of the reviewers are shown in the following with italicized font, the authors' responses and actions taken are shown in non-italicized font, and modified text in the revised manuscript is highlighted in light grey. All line numbers in the comments refer to the revised manuscript (without tracked changes on) and are highlighted in yellow to easily locate them in this document.

ADDRESSING COMMENTS FROM REVIEWER #1:

Comment 1: *The research under review provides a sound assessment of the feasibility of hydrokinetic energy potential of rivers in remote communities of the Global Artic. The study starts with a detaled case study of the Iqaluit Kuunga River in Nunavut, Canada, then upscales the methodology for Canada and 8 countries / regions in the Global Artic.*

The work is original, relevant and has a significant contribution through a thorough analysis of technical and geo-spatial conditions to deploy hydrokinetic energy instalations in rivers, accross remote areas. The significance of the study lays not only in the multi-scale analysis of potentials, but also the outline of chalenges, needs and benefits for the implementation of a local renewable energy grid.

The discussion points and conclusions of the study are supported by results.

I could not identify flaws in the data analysis and interpretation. The methodology is coherent, clear and provides a complete work flow for replicability.

I have no further comments or indications for changing the manuscript's content.

Response: Thank you very much for your comment and review of the manuscript.

ADDRESSING COMMENTS FROM REVIEWER #2:

Reviewer #2 general comments: *Overall, the authors present an interesting and timely study of in-stream renewable energy potential for remote communities in the Arctic based on hydrokinetic energy from field collected data and existing datasets for Canadian rivers. Such an analysis can provide valuable insights into energy transitions and resilience for remote communities that often rely on fossil fuel generation. The research evaluated the technical and socioeconomic feasibility of hydrokinetic energy through a single case study, a global analysis, and an examination of readiness for this technology based on socioeconomic status of various remote communities. Below are my suggestions and comments.*

Major Comment 1: *Methodology: The study leverages a four-tiered approach to evaluate the feasibility of HKE in remote Arctic communities. While the approach is outlined logically, it raises some concerns regarding its robustness. A key limitation to this study is that field collected data at a single site was used to calibrate results for HKE potential across Canada. Because river morphology, flow regimes, and land-use contexts vary widely, it is unclear whether this single-site calibration is transferable to other settings. This approach risks overfitting the model to local conditions rather than demonstrating broad applicability. At minimum, calibration and validation should rely on independent datasets, preferably from different river systems, and the authors should justify why the selected site is representative of the broader population of rivers. A cross-site validation or transferability analysis would also be valuable. If such analyses are not feasible, the manuscript should temper its broader claims accordingly.*

Response: Thank you for your comment. The authors agree additional information on the calibration of the products and data used in this analysis would be useful as well as additional validation locations. Each of the inputs to the estimation of river hydrokinetic energy (river flow, depth, and width) were calibrated separately, and the derived estimates of river hydrokinetic energy were validated at three separate river sites: the Iqaluit Kuunga River, the Riviere-Rouge, and the Spanish River. To increase the number of river systems used in the validation and to improve the validation beyond just Canadian context, three additional validation locations have been added to the manuscript:

(1) The first additional validation location is at Tanana River near Nenana, Alaska, USA at 64.568°N, -149.076°W (Toniolo et al., 2010). This study reported 4,500 W/m^2 for the average power in area of maximum energy in late August when field measurements were taken. (Toniolo et al., 2010). Converting to comparable values by using monthly AEP values, as power in Tanana River wasn't reported as AEP and rather as power density, an estimate of **3.87** MWh/month/m^2 was derived from our study at this river location while model calibrated with ADCP measurements (Toniolo et al., 2010) reported **4.84** MWh/month/m^2.

(2) The second additional validation location is at Kvichak River near Igiugig, Alaska, USA at 59.324°N, -155.915°W The annual power output, depending on siting of the device, was reported to be between 150.28 or 222.59 MWh. (McCallum et al., 2021). Converting to comparable values (9 m^2 capture area for one ORPC RivGen turbine), the modelled energy in the Kvichak River was **24.7** MWh/year/m^2. The global hydrokinetic energy database developed in this work estimated the hydrokinetic resource to be **23.6** MWh/year/m^2.

(3) The third additional validation location is at Neris River, Lithuania at 55.0687°N, 24.282°E (Punys et al., 2015). The mean power density at Jonava gauge station in a normal year was reported as 0.24 kW/m^2. Converting to comparable values, the AEP for this river location is **2.10** MWh/year/m^2. The global hydrokinetic energy database developed in this work estimated the hydrokinetic resource to be **3.22** MWh/year/m^2.

Discussion has been added to the manuscript on the implications of the validation results and the appropriate use cases for the data derived in this work, based on the validation results.

Additionally, information has been added to the manuscript regarding the calibration performed for each separate input for both the Global hydrokinetic energy estimates and Canadian hydrokinetic energy estimates.

Flow values for the **Global dataset** were derived from the GRADES dataset (Lin et al., 2019). This dataset was calibrated using streamflow characteristics rather than parameter regionalization, resulting in improved model performance even at ungauged locations. Model parameters were calibrated and bias corrected against a machine learning-derived map describing global runoff characteristics with discharge observations from 3,000-4,000 naturalized catchments (Beck et al., 2015). The results were validated at over 14,000 hydrometric gauges with more than three years

of data. This comparison demonstrated the robustness and high accuracy of the derived dataset with most of the 14,000 gauge locations having a percent bias between ±20% (Lin et al., 2019)

Depth values for the **Global dataset** were derived using hydraulic geometry relationships for unregulated channels (Bomhof et al., 2015; Jenkinson and Bomhof, 2012) based on the prediction of bank-full depth from a design flow as originally developed by Leopold and Maddock (1953). These power law-based hydraulic geometry relationships were calibrated using flow and depth measurements from hydrometric gauge stations across Canada. They were validated using a jackknife (or leave-one-out) approach for each hydrometric station and showed good agreement in regions in steep or bedrock-based channels (R^2 = 0.73, 0.76) and poorer agreement in regions with more gradual gradients and complex hydrology (R^2 = 0.57, 0.30).

Width values for the **Global dataset** were derived from the GRWL dataset. This dataset was derived from Landsat TM, ETM+, and OLI scenes acquired at approximated months of mean discharge conditions (Allen and Pavelsky, 2018). The modified normalized difference water index (MNDWI) was used with a dynamic thresholding procedure (Li and Sheng, 2012) to create surface water maps, and river centerlines and widths were extracted using the RivWidth algorithm (Pavelsky and Smith, 2008). Visual inspection was used to calibrate the threshold and correct for any gaps, while validation was performed at 1,250 USGS stream gauge locations resulting in a RMSE of 25.2 m (Allen and Pavelsky, 2018).

Flow values for the **CRHE dataset** were derived from two sources: Jenkinson and Bomhof (2014, 2012) and Teufel and Sushama (2022). The flow data from Jenkinson and Bomhof (2014) was derived from hydrometric gauge station data and used a multiple regression with Canonical Correlation Analysis (MR-CCA) to statistically group similar hydrometric stations. The approach was calibrated by adjusting the chi-squared distance value (alpha) to minimize the average of RMSE flow values. These flows were validated using the jackknife or leave-one-out approach and showed good agreement with measured values (R^2 = 0.95). The flow data from Teufel and Sushama (2022) was derived from downscaling hydrological data and converting runoff estimates into streamflow via the WATROUTE routing scheme. The data was validated visually by comparing hydrographs of six hydrometric gauge locations, showing good agreement (Bhattacharjee et al., 2025).

Depth values for the **CRHE dataset** were derived from the same hydraulic geometry relationships for unregulated channels as those used in the Global assessment (Bomhof et al., 2015; Jenkinson and Bomhof, 2012).

Width values for the **CRHE dataset** were derived from Sentinel-2 multispectral satellite imagery and the method used to create surface water maps was Automated Water Extraction Index, No Shadow while the river widths were measured using an automated GIS model (Kirby et al., 2026). This remote sensing-based approach to measuring river widths was calibrated extensively using four machine learning approaches and five spectral index approaches in four geographically diverse locations in two different seasonal conditions (Kirby et al., 2024a, 2024b). Calibration was also performed using sensitivity analysis of thresholds. The calibration process for river width measurements in for the CRHE database is described fully in Kirby et al. (2024a). Validation was performed at 35 geographically diverse river locations in different flow conditions (total of 156 measurements used for validation) resulting in an R^2 of 0.95.

The estimates of hydrokinetic energy resources in the CRHE database were validated extensively in Kirby et al. (2026).

Action taken: Figure 6 was revised to include the additional three validation locations.

Lines 984-990: "(b) Validation of the HKE estimates from the CRHE database and the global database at three locations where the AEP was modelled using field measurements and hydrodynamic models and another three locations from independent hydrokinetic resource assessment studies [26,92,93]. Due to the limited data collection for the study on the Tanana River, Alaska [92], AEP for this location is presented as a monthly value ($MWh/month/m^2$ rather than $MWh/year/m^2$) to provide comparable values."

Lines 494-524: "The river HKE estimates in the CRHE were derived using a multi-step methodology. Discharge estimates were extracted from two datasets. The first dataset consisted of modelled flows in southern Canada obtained by multiple linear regression and canonical correlation analysis informed by Water Survey of Canada flow records to group statistically similar hydrometric data [74]. The approach was calibrated by adjusting the chi-squared distance value (alpha) to minimize the average of RMSE flow values. These flows were validated using the jackknife or leave-one-out approach and showed good agreement with measured values (R^2 =

0.95). The second river flow dataset consisted of modelled flows in northern Canada obtained by downscaling hydrological data and converting runoff estimates into streamflow via the WATROUTE routing scheme [75]. The data was validated visually by comparing hydrographs of six hydrometric gauge locations, showing good agreement [76].

River depth was estimated from hydraulic geometry relationships [74]: power law-based relationships between river depth and river flow that were derived at Water Survey of Canada hydrometric gauge stations [74,77]. These hydraulic geometry relationships were created for unregulated channels [78,79] based on the prediction of bank-full depth from a design flow as originally developed by Leopold and Maddock [80]. They were validated using a jackknife (or leave-one-out) approach for each hydrometric station and showed good agreement in regions in steep or bedrock-based channels (R^2 = 0.73, 0.76) and poorer agreement in regions with more gradual gradients and complex hydrology (R^2 = 0.57, 0.30).

River width was extracted from Sentinel-2 multispectral satellite imagery [35]. The method used to create surface water maps was Automated Water Extraction Index, No Shadow, and the river widths were measured using an automated GIS model [35]. This remote sensing-based approach to measuring river widths was calibrated extensively using four machine learning approaches and five spectral index approaches in four geographically diverse locations in two different seasonal conditions [81,82]. Calibration was also performed using sensitivity analysis of thresholds. The calibration process for river width measurements for the CRHE database is described fully in Kirby et al. [81]. Validation was performed at 35 geographically diverse river locations in different flow conditions (total of 156 measurements used for validation) resulting in an R^2 of 0.95 [35]."

Lines 548-617: "Each of the inputs to the estimation of river HKE (river flow, depth, and width) were calibrated separately in the works that they were acquired from. The derived estimates of river HKE were validated at six separate river sites: three Canadian sites in which full HKE resource estimations were performed according to the IEC [52] standards by the authors and three other independent, international sites where HKE resources were estimated. The three independent river sites were not necessarily performed according to the IEC [52] standards, yet they still provide a useful metric for comparison to the globally derived HKE estimates.

The global Arctic HKE estimates were derived using a methodology consistent with the IEC [52] standards for river HKE resource estimation, wherein flow, velocity, and power duration curves

were developed to compute AEP for each river cross section. This is a similar methodology that was applied to the Iqaluit Kuunga River case study. To generate the FDCs, the Global Reach-level a priori Discharge Estimates (GRADES) dataset [84] was used which contains model-derived daily discharge estimates for 1980-2013 at a global scale. The GRADES dataset was calibrated using streamflow characteristics rather than parameter regionalization, resulting in improved model performance even at ungauged locations [85]. Model parameters were calibrated and bias corrected against a machine learning-derived map describing global runoff characteristics with discharge observations from 3,000-4,000 naturalized catchments [86]. The results were validated at over 14,000 hydrometric gauges with more than three years of data. This comparison demonstrated the robustness and high accuracy of the derived dataset with the majority of the 14,000 gauge locations having a percent bias between ±20% [84]. The FDCs from the GRADES data were spatially coupled to river locations using MERIT hydro streamlines [87].

River channel geometry was estimated from two sources: river depth from the same hydraulic geometry relationships used in the CRHE database [35,74] and river width from the Global River Widths from Landsat (GRWL) dataset [88]. The GRWL dataset includes estimates of river width at mean annual flow with an average spatial resolution of 35 m. This dataset was derived from Landsat TM, ETM+, and OLI scenes acquired at approximated months of mean discharge conditions [88]. The modified normalized difference water index (MNDWI) was used with a dynamic thresholding procedure [89] to create surface water maps, and river centerlines and widths were extracted using the RivWidth algorithm [90]. Visual inspection was used to calibrate the threshold and correct for any gaps, while validation was performed at 1,250 USGS stream gauge locations resulting in a RMSE of 25.2 m [88].

For calculation of velocity and HKE, a rectangular channel shape was assumed. VDCs were derived for each cross section by estimating the cross-sectional mean velocity for each FDC exceedance probability by conservation of mass, using the derived river depth at each flow exceedance and a constant river width at mean annual flow. Because multiple width measurements across different flow conditions are not available globally in the GRWL dataset, as they are in the CRHE database, estimates of velocity at high exceedance and low exceedance probabilities on the VDC are likely to contain some error. The power at each exceedance was calculated using

Equation 1, assuming a turbine cross-sectional area of 1 m^2 for standardization across sites. The resulting PDCs were interpolated using Equation 2 to compute the AEP at each river cross section.

The global Arctic HKE estimates were validated at locations where measurements of river HKE had been previously derived using a combination of field measurements (usually using ADCP) and numerical modelling. Three validation locations were provided by the authors and three additional validation locations were acquired from independent literature. The three Canadian validation river sites provided by the authors were the Iqaluit Kuunga River (63.787°N, -68.622°W), the Riviere-Rouge (45.645°N, -74.691°W) [69], and the Spanish River (46.177°N, -82.091°W) [91]. Further details on these validation locations is available in [35].

The three independent, international validation locations were the Tanana River near Nenana, Alaska USA (64.568°N, -149.076°W) [92], the Kvichak River near Igiugig, Alaska, USA (59.324°N, -155.915°W) [26], and the Neris River near Jonava, Lithuania (55.0687°N, 24.282°E) [93]. The HKE study on the Tanana River reported 4,500 W/m^2 for the average power in area of maximum energy in late August when field measurements were taken [92]. Converting to comparable values by using monthly AEP values, as power in Tanana River wasn't reported as AEP and rather as power density, an estimate of 3.87 MWh/month/m^2 was derived from our study at this river location while model calibrated with ADCP measurements reported 4.84 MWh/month/m^2 [92]. The HKE study on the Kvichak River reported the annual power output, depending on siting of the device, to be between 150.28 or 222.59 MWh [26]. Converting to comparable values, using a 9 m^2 capture area for one ORPC RivGen turbine, the modelled energy in the Kvichak River was 24.7 MWh/year/m^2. The global HKE database developed in this work estimated the hydrokinetic resource to be 23.58 MWh/year/m^2. The HKE study on the Neris River reported a mean power density of 0.24 kW/m^2 at Jonava gauge station in a normal year [93]. Converting to comparable values, the AEP for this river location is 2.10 MWh/year/m^2. The global HKE database developed in this work estimated the hydrokinetic resource to be 3.22 MWh/year/m^2."

Lines 298-311: "The validation of the global Arctic HKE estimates at six geographically diverse river sites revealed that the global river HKE dataset, similar to the CRHE database, is good at identifying feasible communities for further HKE resource investigation. The use cases for these estimates include identification of feasible communities, examination of the proximity of communities to river sites with favourable resource potential, and community-to-community

resource comparison for energy decision-making. The average difference between modelled AEP and estimates derived from the global dataset was 30.5% (ranging from 4.5% to 53.3%), indicating that the dataset provides a good order-of-magnitude estimate rather than a precise quantification of HKE potential. Given that the required field data collection following IEC [52] standards of river HKE estimation (see Methods) is costly and the required hydrodynamic modelling can be time-intensive, such preliminary estimates are valuable for guiding these subsequent, resource-intensive analyses. Overall, the global Arctic HKE dataset provides a strong foundation for targeted HKE investigation and development."

Major Comment 2: *Methodology: Feedback on river HKE development and broader renewable energy transition efforts in Arctic regions were collected through in-situ community engagement during field work near Iqaluit, Nunavut Canada and through structured knowledge dissemination events involving local residents. It is not clear from the methods the protocol used to collect such data and the transferability of these results to the broader Arctic community. Please provide additional details regarding questions asked, number of respondents, characteristics of respondents, and literature clarifying the approach used in the context of the study and broader social science methods.*

Response: Thank you for your comment. The methods and protocol used to collect the themes of socioeconomic factors related to renewable energy transition in northern communities have been added to the manuscript, specifically in Table 1 and in the Methods and Discussion sections.

Ethical clearance to perform this work was granted by the Nunavut Research Institute, the Research Ethics Board of the University of Ottawa, and the Amaruq Hunters and Trappers Association. Proper ethical clearance was only possible through local community member participation via collaborators at Polar Outfitting in Iqaluit, Nunavut.

The rationale for the use of an unstructured and semi-structured approach was based on expert opinion (i.e., professionals with many years of experience working with and in Arctic and remote communities) regarding effective methods for gathering information in northern communities. A formal interview structure originally did not yield strong engagement; one pilot using formal questions was conducted with poor results. As a result, the approach was changed to unstructured

and semi-structured interviews, as originally suggested by expert opinion. Informal and semi-structured approaches allow for greater flexibility. This approach also allowed for sensitivity and caution in establishing an ethical relationship between the interviewee and the researcher.

The dissemination sessions, interviews, and discussions were not transcribed. Rather, generalized points, ideas, and themes were collected instead of specific wording.

Interviews are more commonly performed in the Western Arctic to gather socio-economic information and to understand the knowledge, values, beliefs, and decision-making processes of stakeholders (Young et al., 2018). As such, literature examining renewable energy trends in the Arctic may exhibit a Western bias. Possible improvements to the approach used in this study include the implementation of interview coding.

Actions taken: Lines 220-224: "Unstructured and semi-structured events were conducted to collect data regarding socioeconomic barriers to renewable energy transition in the global Arctic. This data was used to identify emerging patterns for barriers, requirements, and benefits of renewable energy transition. More information on these unstructured and semi-structured methods are available in the Methods section."

Lines 369-378: "Because the data related to renewable energy socioeconomic factors in northern communities was collected mostly at Canadian locations, a heavy Canadian bias is likely present, specifically in the socioeconomic analysis of this study. Additionally, interviews are more commonly performed in the Western Arctic to gather socio-economic information and to understand the knowledge, values, beliefs, and decision-making processes of stakeholders [68]. As such, the literature examining renewable energy trends in the Arctic may also exhibit a Western bias. Future work could focus on the examination of Global renewable energy socioeconomic factors to reduce such biases. Possible improvements to the approach used in this study include the implementation of interview coding."

Lines 619-647: "Feedback on river HKE development and broader renewable energy transition efforts in Arctic regions was collected through in-situ community engagement during fieldwork near Iqaluit, Nunavut, Canada, and via semi-structured knowledge dissemination events involving local residents and key informants. Ethical clearance to perform this work was granted by the Nunavut Research Institute, the Research Ethics Board of the University of Ottawa, and the

Amaruq Hunters and Trappers Association. Proper ethical clearance was only possible through local community member participation via collaborators at Polar Outfitting in Iqaluit, Nunavut. The authors gathered data regarding barriers to renewable energy transitions in northern communities, the general interest in renewable energy systems, and if the participants expected any benefits from renewable energy in their communities. The participants were also asked if there were any foreseeable challenges to HKE deployment in their communities. Participants were asked open- and closed-ended questions to elicit insights on institutional, financial, technical, and logistical constraints to renewable energy deployment. Table 1 presents the community engagement and structured dissemination events undertaken from which the socioeconomic data was collected.

The approach employed in this study was an unstructured and semi-structured approach to information gathering. The rationale for the use of an unstructured and semi-structured approach was based on expert opinion (i.e., professionals with many years of experience working with and in Arctic and remote communities) regarding effective methods for gathering information in northern communities. A formal interview structure originally did not yield strong engagement; one pilot using formal questions was conducted with poor results. As a result, the approach was changed to unstructured and semi-structured interviews, as originally suggested by expert opinion. Informal and semi-structured approaches allow for greater flexibility. This approach also allowed for sensitivity and caution in establishing an ethical relationship between the interviewee and the researcher. Additionally, the dissemination sessions, interviews, and discussions were not transcribed. Rather, generalized points, ideas, and themes were collected instead of specific wording."

Lines 1003-1005: "Table 1: Stakeholder and representative groups from which data on barriers, requirements, and benefits of Arctic renewable energy transition were collected.

[See Table 1 in the manuscript]"

Major Comment 3: *Introduction: Limitations to this study include optimal sizing of HKE projects, reliability of HKE, and policy support. However, there is a broad and growing literature on the potential impacts of renewable energy development on ecosystems and wildlife. Please*

either include an analysis of the potential impacts HKE could have on water quality, habitat structure, sediment dynamics, and freshwater biotic communities, or include a dedicated discussion of the potential ecological implications of such development.

Response: Thank you for your comment. Information on the impact hydrokinetic turbines have on local hydrodynamics and sediment transport has been added to the Discussion section of the manuscript. Additionally, potential impacts to river ecology and aquatic species have been added, building on the observed impacts turbines have on hydrodynamics and channel structure. Following this discussion, recommendations are made to minimize turbine impact based on the current literature.

Actions taken: Lines 312-360: "There is a growing body of literature examining the potential impacts of riverine HKE development on local hydrodynamics and sediment transport, and the resulting implications for aquatic ecosystems and wildlife. In general, turbines alter local flow conditions and sediment transport processes, although the magnitude and spatial extent of these effects depend on several key parameters. These include turbine type and size, often represented by tip speed ratio (TSR), where higher TSR typically results in a more localized wake and faster recovery [53,54]; ambient turbulence and flow velocity, with higher turbulence promoting faster wake recovery [55,56]; and the presence of debris, which can increase turbulence near the bed, strengthen scour, and accelerate wake recovery [57]. Turbine design modifications can also influence wake structure. For example, turbine winglets may reduce near-wake vortices while strengthening far-wake vortices [58]. Additional factors include rotor proximity to the bed, which can intensify localized scour [59], and flow depth, where deeper flows tend to allow faster velocity recovery but slower recovery of turbulence intensity [60]. Sediment grain size appears to have little influence on wake recovery [53]. Reported turbine wake recovery distances generally range from approximately 4D to more than 15D downstream [53–55,57–59,61], highlighting the varied influence these parameters have on the extent of the turbine's hydrodynamic impact. Increased turbulence intensity within the wake can lead to localized scour and downstream deposition, linking hydrodynamic changes directly to sediment transport processes. Conversely, sediment scour can further influence hydrodynamics by increasing turbulence and mixing, which may accelerate wake recovery [53].

Because hydrokinetic turbines are known to generate localized scour, the immediate hyporheic zone may be altered and this may increase hyporheic exchange depending on the depth of scour

[62]. The hyporheic zone provides habitat for diverse assemblages of macroinvertebrates, meiofauna, microorganisms, and various life stages of aquatic organisms [63]. These environments support interconnected chemical, physical, and biological processes that play an important role in river ecosystem functioning [64]. As a result, balancing efficient turbine operation, optimized turbine staging, and protection of river ecology is important. Potential strategies include maintaining sufficient distance between the rotor and the riverbed, optimizing the TSR, and designing turbines to minimize debris accumulation. Despite these localized effects, sediment transport impacts associated with hydrokinetic turbines are considerably smaller than those caused by dam-based hydropower [20,21]. Consequently, hydrokinetic turbines are generally regarded as a relatively environmentally friendly renewable energy technology [65].

Because turbines also produce measurable changes in local hydrodynamics, it is important to consider their potential influence on fish behaviour. Current literature generally indicates that hydrokinetic turbines pose relatively low risk to fish movement, as fish passage was not significantly affected and avoidance behaviour was commonly observed across a variety of conditions [66]. Field observations from a turbine installation in the Kvichak River near Igiugig, Alaska monitored Sockeye Salmon smolt interactions over a 20-day period and found that blade strike events were rare, although some smolts exhibited temporary disorientation due to turbine-induced hydraulic effects [67]. These findings suggest that while impacts appear limited, additional research is needed to fully understand hydrokinetic turbine interactions with fish. Design guidelines have been proposed to further reduce ecological risk by limiting factors such as physical strike, shear, pressure changes, and excessive turbulence. These guidelines include, but are not limited to, operating turbines at low rotational speeds (around 20 RPM), maintaining low blade tip velocities (below 5 m/s), and maximizing spacing between multiple turbines [65]."

Minor Comment 1: *Line 54 – consider including dollars in parenthesis when reporting international currency's; 3 rubles per kWh (x $ per kWh)*

Response: Thank you for your comment. The manuscript was revised to include dollars for comparison.

Actions taken: Lines 59-61: "Similarly, in Arctic Russia, monopolies and high electricity prices strain households financially, where prices reach 600 rubles (~7.56 USD) per kWh compared to the national average of 3 rubles per kWh (~0.04 USD) [4]."

Minor Comment 2: *Line 485 – The CRHE dataset contains estimates of KHE for all rivers in Canada exceeding 100 m3/s and are greater than 20 m in width. Do these parameters reflect the in-situ river used for calibration and validation?*

Response: Thank you for your comment. Yes, all rivers used in the validation of both the CRHE database and the Global HKE database were performed on rivers with greater than 100 m^3/s flow and 20 m width. This detail has been added to the manuscript.

Actions taken: Lines 491-493: "The CRHE database includes estimates of HKE for all rivers in Canada that can exceed 100 m^3/s at any point in their hydrological cycle and are greater than 20 m in width, and has been comprehensively validated with rivers of similar characteristics."

Minor Comment 3: *Line 37 through 40 – what is the relative risk caused by these changes?*

Response: Thank you for your comment. The risk of changing sea ice patterns and its impact on subsistence activities in northern communities has not yet been quantified. Studies that have quantified the impact of black carbon on snow and ice albedo have approximated a 20% albedo reduction during glacier melt season in the Tibetan Plateau (Kang et al., 2020). Despite the lack of quantified risk in literature, these changes are common knowledge in northern communities (Bishop et al., 2025; Simonee et al., 2021), with climate adaptation tools – such as SmartICE (2026) – being developed to aid hunters to travel on sea ice safely. This information has been added to the manuscript.

Actions taken: Lines 37-47: "All Arctic regions, reliant on seasonal navigation and winter roads, face logistical challenges and negative consequences of delivering diesel [4]. Black carbon from fossil fuels accelerates local ice and snow melt, reducing the Arctic's reflectivity [4,5] and altering historical ice stability patterns. These changes lead to increased risk for hunters traveling to traditional hunting areas. The risk of changing sea ice patterns and its impact on subsistence

activities in northern communities has not yet been quantified. Studies that have quantified the impact of black carbon on snow and ice albedo have approximated a 20% albedo reduction during glacier melt season in the Tibetan Plateau [6]. Despite the lack of quantified risk in literature, these changes are common knowledge in northern communities [7,8], with climate adaptation tools – such as SmartICE [9] – being developed to aid hunters to travel on sea ice safely."

Minor Comment 4: *Line 31 through 33 – Does this statistic include European Arctic communities?*

Response: Thank you for your comment. No, this statistic only includes Canada, Greenland, and Alaska (North American Arctic communities). This has been clarified in the manuscript.

Actions taken: Lines 32-34: "As of 2021, 79% of Arctic communities in North America (Canada, Greenland, and Alaska, USA) still relied on diesel for electricity [2]."

Minor Comment 5: *When necessary, include text directing the reader to the methods.*

Response: Thank you for your comment. The authors agree this will be helpful to direct the reader when more details are available in the methods. The manuscript has been revised as appropriate.

Actions taken: Line 111: "Further information on the field data collection and processing is available in the Methods."

Lines 144-145: "More details on this process are provided in the Methods section."

Lines 223-224: "More information on these unstructured and semi-structured methods are available in the Methods section."

References:

Allen, G.H., Pavelsky, T.M., 2018. Global extent of rivers and streams. Science 361, 585–588. https://doi.org/10.1126/science.aat0636

Beck, H., Roo, A., van Dijk, A., 2015. Global Maps of Streamflow Characteristics Based on Observations from Several Thousand Catchments. J. Hydrometeorol. 16, 1478–1501. https://doi.org/10.1175/JHM-D-14-0155.1

Bhattacharjee, K., Sushama, L., Cousineau, J., 2025. Hydrokinetic resource assessment for the Canadian Arctic for turbine-based power generation. J. Water Clim. Change 16, 1238–1259. https://doi.org/10.2166/wcc.2025.353

Bishop, B., Paquette, E., Carter, N., Ljubicic, G., Oliver, E.C.J., Aporta, C., 2025. Inuit uses of weather, water, ice, and climate indicators to assess travel safety in Arctic Canada, Alaska, and Greenland: a scoping review. FACETS 10, 1–25. https://doi.org/10.1139/facets-2024-0107

Bomhof, J., Rennie, C.D., Jenkinson, R.W., 2015. Use of Local Soil and Vegetation Classifications to Improve Regional Downstream Hydraulic Geometry Relations. J. Hydraul. Eng. 141, 04014090. https://doi.org/10.1061/(ASCE)HY.1943-7900.0000978

Jenkinson, W., Bomhof, J., 2014. Assessment of Canada's Hydrokinetic Power Potential. Phase III Report: Resource Estimation.

Jenkinson, W., Bomhof, J., 2012. Assessment of Canada's Hydrokinetic Power Potential: Phase II Report Methodology Validation.

Kang, S., Zhang, Y., Qian, Y., Wang, H., 2020. A review of black carbon in snow and ice and its impact on the cryosphere. Earth-Sci. Rev. 210, 103346. https://doi.org/10.1016/j.earscirev.2020.103346

Kirby, K., Ferguson, S., Rennie, C.D., Cousineau, J., Burcher, R., Nistor, I., 2026. High-resolution mapping of river hydrokinetic energy resources in Canada using remote sensing. Renew. Energy 256, 123970. https://doi.org/10.1016/j.renene.2025.123970

Kirby, K., Ferguson, S., Rennie, C.D., Cousineau, J., Nistor, I., 2024a. Identification of the best method for detecting surface water in Sentinel-2 multispectral satellite imagery. Remote Sens. Appl. Soc. Environ. 101367. https://doi.org/10.1016/j.rsase.2024.101367

Kirby, K., Rennie, C.D., Poot, R., Ferguson, S., Cousineau, J., Nistor, I., 2024b. Accuracy of Surface Water Maps Derived from Radar Satellite Imagery Compared to Multispectral Satellite Imagery. Can. J. Remote Sens. 50, 2433591. https://doi.org/10.1080/07038992.2024.2433591

Leopold, L., Maddock, T., 1953. The hydraulic geometry of stream channels and some physiographic implications. U.S. Government Printing Office, Washington, D.C.

Li, J., Sheng, Y., 2012. An automated scheme for glacial lake dynamics mapping using Landsat imagery and digital elevation models: a case study in the Himalayas. Int. J. Remote Sens. 33, 5194–5213. https://doi.org/10.1080/01431161.2012.657370

Lin, P., Pan, M., Beck, H.E., Yang, Y., Yamazaki, D., Frasson, R., David, C.H., Durand, M., Pavelsky, T.M., Allen, G.H., Gleason, C.J., Wood, E.F., 2019. Global Reconstruction of Naturalized River Flows at 2.94 Million Reaches. Water Resour. Res. 55, 6499–6516. https://doi.org/10.1029/2019WR025287

McCallum, C.S., Kumar, N., Curry, R., McBride, K., Doran, J., 2021. Renewable electricity generation for off grid remote communities; Life Cycle Assessment Study in Alaska, USA. Appl. Energy 299. https://doi.org/10.1016/j.apenergy.2021.117325

Pavelsky, T.M., Smith, L.C., 2008. RivWidth: A Software Tool for the Calculation of River Widths From Remotely Sensed Imagery. IEEE Geosci. Remote Sens. Lett. 5, 70–73. https://doi.org/10.1109/LGRS.2007.908305

Punys, P., Adamonyte, I., Kvaraciejus, A., Martinaitis, E., Vyciene, G., Kasiulis, E., 2015. Riverine hydrokinetic resource assessment. A case study of a lowland river in Lithuania. Renew. Sustain. Energy Rev. 50, 643–652. https://doi.org/10.1016/j.rser.2015.04.155

Simonee, N., Alooloo, J., Carter, N.A., Ljubicic, G., Dawson, J., 2021. Sila Qanuippa? (How's the Weather?): Integrating Inuit Qaujimajatuqangit and Environmental Forecasting Products to Support Travel Safety around Pond Inlet, Nunavut, in a Changing Climate. https://doi.org/10.1175/WCAS-D-20-0174.1

SmartICE, 2026. SmartICE Monitoring & Information Inc. [WWW Document]. SmartICE SmartICE. URL https://smartice.org/ (accessed 3.17.26).

Teufel, B., Sushama, L., 2022. Hydrokinetic resource estimation for the Canadian Arctic – Phase I (Technical Report). Department of Civil Engineering and Trottier Institute for Sustainability in Engineering and Design, McGill University.

Toniolo, H., Duvoy, P., Anlesberg, S.V., Johnson, J., 2010. Modelling and field measurements in support of the hydrokinetic resource assessment for the Tanana river at Nenana, Alaska. Proc. Inst. Mech. Eng. Part J. Power Energy 224, 1127–1139. https://doi.org/10.1243/09576509JPE1017

Young, J.C., Rose, D.C., Mumby, H.S., Benitez-Capistros, F., Derrick, C.J., Finch, T., Garcia, C., Home, C., Marwaha, E., Morgans, C., Parkinson, S., Shah, J., Wilson, K.A., Mukherjee, N., 2018. A methodological guide to using and reporting on interviews in conservation science research. Methods Ecol. Evol. 9, 10–19. https://doi.org/10.1111/2041-210X.12828
